# Post synthetic modification of Cu-MOF with riboflavin for stable electrocatalytic conversion of CO_2_ to formate

**DOI:** 10.1039/d6ra05541a

**Published:** 2026-11-10

**Authors:** Pandiaraj Sekar, Punitharaj Vasanthakumar, Selvaraj Paramasivam, Rajasekharan Jayakumari Deepak, Laura Calvillo, Sunandan Sarkar, Nattamai Bhuvanesh, Kasthuri Annamalai Sami, Shanmugam Senthil Kumar, Ramasamy Karvembu

**Affiliations:** a Department of Chemistry, National Institute of Technology Tiruchirappalli 620015 India kar@nitt.edu; b Electrodics and Electrocatalysis Division, CSIR-Central Electrochemical Research Institute Karaikudi 630006 India ssenthilkumar@cecri.res.in; c Department of Chemical Sciences, University of Padova Via F. Marzolo 1 35131 Padova Italy laura.calvillolamana@unipd.it; d Department of Chemistry, Texas A & M University College Station Texas 77842 USA; e Department of Physics, American University of Sharjah Sharjah 26666 United Arab Emirates

## Abstract

The major challenges in the development of metal–organic framework (MOF)-based electrocatalysts for CO_2_ reduction reaction (CO_2_RR) are related to their electrochemical stability, product selectivity, and electrical conductivity. To overcome these, we prepared a stable MOF-based electrocatalyst *via* surface functionalization of a Cu-MOF with the redox-active riboflavin (Rbf) molecule using a mechanochemical method. The modified catalyst, Rbf–Cu-MOF (1 : 1), showed three-fold higher electrocatalytic activity in the reduction of CO_2_ to formate than that of pristine Cu-MOF. Rbf–Cu-MOF showed the highest faradaic efficiency (FE) of 90% at −1.1 V (*vs.* RHE), and formate was the primary product in the reduction reaction. Post-catalytic analysis [High resolution transmission electron microscopy (HR-TEM) and X-ray absorption near-edge structure (XANES)] of the Cu-MOF samples, with and without Rbf, revealed that the Cu-MOF tended to irreversibly form Cu nanoparticles under the highly reductive electrochemical environment, while Rbf–Cu-MOF remained structurally stable for 100 h and catalytically active throughout the reaction, which can be attributed to the existence of Rbf. These findings provide vital information for employing MOF-based materials for electrocatalysis and other energy-related applications.

## Abstract 2

Post-synthetic modification of a Cu-MOF with riboflavin enhances the structural stability of the MOF under harsh electrochemical conditions and improves the electrocatalytic CO_2_ conversion.

## Graphical abstract

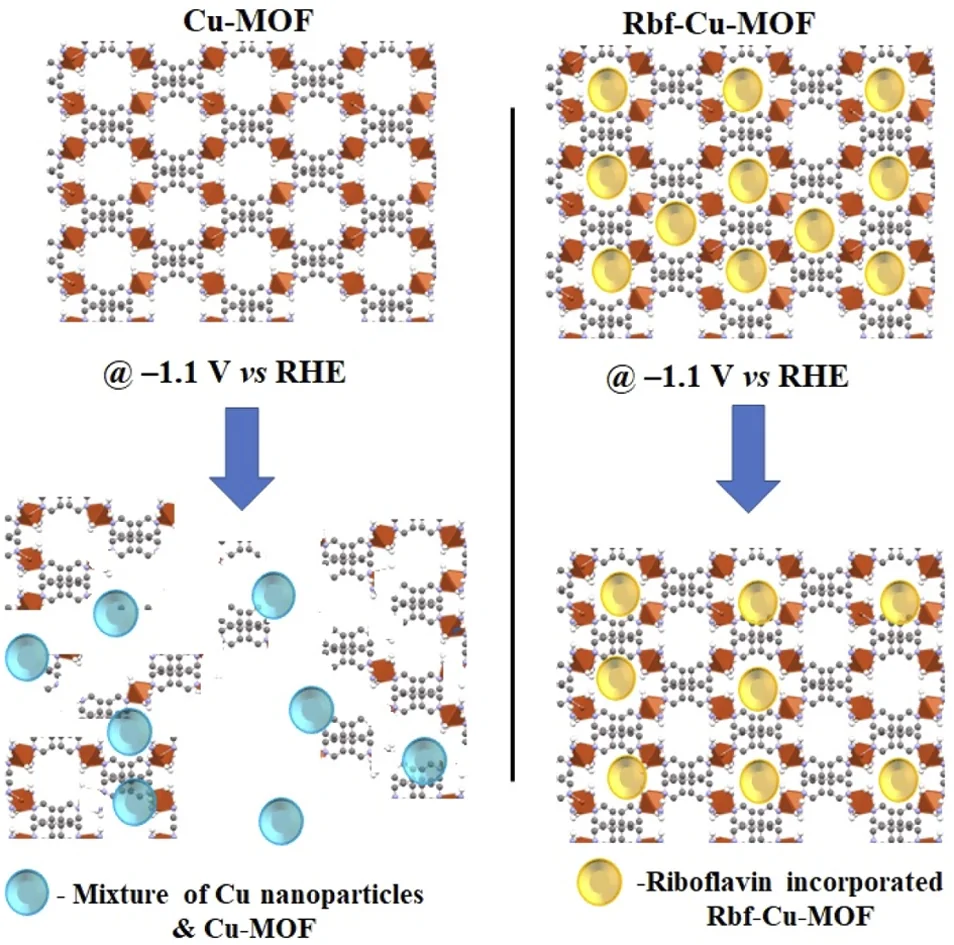

## Introduction

The electrocatalytic reduction of CO_2_ (CO_2_RR) into value-added feedstocks triggered by green energy-based electricity is an important approach for promoting CO_2_ recycling and hence, reducing the CO_2_ level in the atmosphere.^1–3^ This emerging technology can help to achieve the targets of the “Paris Agreement” and strengthen the United Nations' goal of climate action [Sustainable Development Goal (SDG)-13].^4^ To date, numerous research projects focusing on the electrocatalytic reduction of CO_2_ to formate, alcohols, or other hydrocarbons have been carried out because of its quick and straightforward chemical pathway.^5–8^ Despite these advantages, the commercial viability of CO_2_RR mainly depends on long-term operational stability, while maintaining high faradaic efficiency (FE) over the extended duration of electrolysis.^9^

Metal–organic frameworks (MOFs) are well-known for their chemical versatility, wide surface area, tunable pores, and abundant catalytic centers. The uniform arrangement of active metal ions in the MOF skeleton improves its electrocatalytic activity (particularly, CO_2_ and oxygen reduction reactions),^10–12^ since it offers accessible pathways for mass transfer, enhancing the overall catalytic activity and FE.^13,14^ At the molecular level, the metal nodes connected through the ligands form the catalytically active centers. For instance, Shimoni *et al.* prepared an Fe-porphyrin MOF and applied it as an electrocatalyst for the reduction of CO_2_ to CO. This research highlights that the Fe atoms bonded with N are the catalytically active centers, and the presence of phenolic groups on the MOF stabilizes the intermediates and enhances the catalytic activity.^15,16^ Despite these advancements, the stability of MOFs under high negative potential remains highly challenging.^17,18^ Recently, Smith *et al.* observed the conversion of HKUST-1 MOF film into a MOF-derived Cu-based material (MOF-*d*-Cu) during the chronoamperometric studies in CO_2_-saturated electrolyte.^19^ Their studies revealed that the conversion of Cu^2+^ ions into metallic Cu, and leaching of the linkers (as an un-metallated ligand) from HKUST-1 into the solution (electrolyte) occurred during the CO_2_ electrolysis. Similarly, Cu-phthalocyanine, a unique metal complex, has been shown to undergo demetallation to form Cu nanoparticles during the CO_2_RR.^20^ The formation of metal clusters raises an important question of whether the intrinsic catalytic activity mainly originates from the MOFs, nanoparticles, or both. MOFs are generally prepared and stable in organic solvents. However, most electrocatalytic reactions (*i.e.*, water splitting, CO_2_RR, and nitrate conversion) occur in aqueous electrolytes. Hence, the incompatibility of MOFs with aqueous electrolytes hinders their utility in electrocatalysis. To realize the intrinsic catalytic activity that primarily originates from the active sites of MOFs, it is crucial to preserve its structural integrity under the electrochemical environments. In addition, MOFs are less conductive; hence, it is quite challenging to fabricate electrode materials from MOFs for electrocatalysis. These issues must be addressed while employing MOFs as potential electrocatalysts for CO_2_ reduction to ensure stable performance and large-scale application.

Recently, post synthetic modification (PSM) has emerged as an attractive method for tuning MOFs towards specific applications; because it can be accomplished under relatively mild conditions while preserving the structural integrity of the parent MOFs.^21^ For example, viologen (a photoactive molecule)-functionalized MOF (MIL-125-RV^2+^) exhibited excellent photocatalytic activity towards the reduction of CO_2_ into formic acid.^22^ Another important study by the Jiang group highlighted that both the stability and catalytic property can be improved by modifying the pore environment of the MOFs. In their work, 2,4-bis(3,5-dicarboxyphenylamino)-6-ol triazine (H_4_BDPO) was used as an organic spacer for the preparation of Cu-MOF, called JUC-1000, in aqueous solution, and it showed an enhanced catalytic activity towards CO_2_ fixation.^23^

Motivated by these studies, we chose riboflavin (Rbf, vitamin B_2_) to modify the Cu-MOF in order to improve its catalytic activity and structural stability. Rbf is well-known for its biological electron transfer reactions and has been extensively used in electrochemical redox reactions.^24^ In our earlier work, Rbf was used for the PSM of Ni-MOF, and the modified MOF was applied as a multifunctional electrocatalyst for oxygen evolution reaction, oxygen reduction reaction and biomass conversion.^25^ The current study represents a significant departure in both material functionality and electrochemical application. Unlike the Ni-based system which focused on oxidation reactions, this Cu-MOF addresses the CO_2_RR. The key innovation lies in the ability of Rbf to suppress the irreversible transformation of Cu-MOF into Cu nanoparticles under the reduction environment, thereby providing high structural stability. This stabilization, combined with enhanced catalytic selectivity, favors efficient CO_2_ to formate conversion.

In this context, we prepared a Cu-MOF in water at 40 °C, and further modified it with Rbf using a simple and facile mechanochemical method to obtain Rbf–Cu-MOF. Without altering the original morphology or structure, the added Rbf molecules created a homogeneous distribution of active sites, tailoring the local microenvironment. The CO_2_ reduction performed in an aqueous NaHCO_3_ electrolyte demonstrated that Rbf–Cu-MOF showed a three-fold higher conversion of CO_2_ to formate compared to the pristine Cu-MOF, at a potential of −1.1 V (*vs.* RHE), along with the maximum FE of 90%, and advantageously, it is stable for 100 h.

## Experimental

### Materials

Copper nitrate trihydrate, 4,4′-bipyridine (4,4′-bpy), and Rbf were obtained from Sigma-Aldrich. The chemicals and reagents were of analytical grade and directly used for our studies.

### Synthesis of Cu-MOF

Cu-MOF was prepared by heating a mixture of copper nitrate trihydrate (60.4 mg, 0.25 mmol) and 4,4′-bpy (156 mg, 1 mmol) in water in an oil bath at 40 °C for 30 min. The resulting solid was then filtered and dried to obtain the blue-colored Cu-MOF (Fig. S1).

### Synthesis of Rbf–Cu-MOF

Cu-MOF surface was functionalized with Rbf molecules using a simple and green mechanochemical method.^25^ Cu-MOF and Rbf were ground with the help of a mortar and pestle to get Rbf–Cu-MOF. The blue color of Cu-MOF changed into green after the addition of Rbf, as shown in Fig. S1. Three Rbf–Cu-MOF samples with different feed molar ratios of Cu-MOF and Rbf were prepared to optimize the contribution of both components. With this aim, the concentration of Cu-MOF (1 mmol) was fixed, and that of the Rbf was varied from 0.5 to 2 mmol to ensure the optimal addition of Rbf onto the Cu-MOF.

### Physicochemical characterization

Scanning electron microscopy (SEM) images were obtained using a Tescan VEGA 3SBH microscope, whereas high-resolution transmission electron microscopy (HR-TEM) and scanning transmission electron microscopy (STEM) coupled with high angle annular dark field (HAADF) analyses were performed using a Thermo Scientific – Tecnai G2 F20 microscope operated at an accelerating voltage of 300 kV. The samples for TEM analysis were prepared by dispersing the solid materials in isopropyl alcohol (IPA), followed by drop casting onto a 200-mesh copper grid coated with a carbon film and dried under a lamp for 12 h. Powder X-ray diffraction (PXRD) measurements were performed using a PANalytical B.V. Empyrean instrument equipped with a Cu Kα source (40 kV, 40 mA), while single-crystal XRD was carried out using a Bruker Quest X-ray diffractometer with a PHOTON III detector. PXRD investigation proceeded over a 2*θ* range from 5° to 80° at a sweep rate of 0.5° min^−1^. X-ray absorption was measured on beamline B18 at Diamond Light Source (UK) with a ring energy of 3 GeV and a current of 300 mA. The monochromator used was Si(111) crystals operating in the Quick EXAFS (QEXAFS) mode. The pellets of commercial copper oxide (Sigma-Aldrich), Cu-MOF and Rbf–Cu-MOF were used for X-ray absorption measurements in transmission mode at the Cu K (8979 eV) absorption edge at 298 K. The X-ray absorption of Cu foil was monitored simultaneously. The monochromator was calibrated using a Cu foil before the measurements. The acquired data were processed and analyzed using the Athena and Artemis programs, which implement the FEFF6 and IFEFFIT codes, respectively. In addition, pellets of the post-catalysis samples (10 h at −1.1 V *vs.* RHE) were measured. X-ray photoemission spectra (XPS) were recorded using a Thermo Scientific ESCALAB 250 Xi XPS spectrometer.

### Electrochemical characterization

Electrochemical analyses were performed using a three-electrode, H-type electrochemical cell comprising a glassy carbon (GC) working electrode, Ag/AgCl (in saturated KCl) reference electrode, and Pt counter electrode. An aqueous NaHCO_3_ (0.5 M) solution was used as an electrolyte. The catalyst slurry was prepared as follows: 5 mg of the prepared catalyst and 1 mg of Vulcan carbon were mixed and transferred to a vial. Further, 1 mL of ethanol–water (3 : 2) solvent and Nafion binder (10 µL) were added to the mixture. Then, the mixture was sonicated for 30 min to get a homogeneous suspension. 5 µL of the catalyst ink was coated on a GC electrode (geometrical area = 0.0706 cm^2^) by drop casting and dried under air. The electrocatalytic activity was evaluated by cyclic voltammetry (CV) and linear sweep voltammetry (LSV) in N_2_- and CO_2_-saturated electrolyte at scan rates of 50 and 10 mV s^−1^. For chronoamperometric measurements, carbon paper (1 × 3 cm^2^) was used as working electrode. 20 µL of the Rbf–Cu-MOF was coated on carbon paper (Toray) and dried for 30 min under an IR lamp. CO_2_ was introduced into the cathode chamber at a rate of 10 mL min^−1^. The CO_2_ electrolysis measurements were performed for more than 100 min at different potentials, ranging from −0.3 to −1.3 V *vs.* RHE. Each electrochemical test was preceded by at least 30 min of N_2_ or CO_2_ bubbling in the electrolyte solution. Long-term durability analysis was performed using chronoamperometry method in a H-type cell, where the anode and cathode chambers were separated by a Nafion membrane (N-117).

### Quantification of formate by NMR (^1^H and ^13^C) analyses

For the quantification of the product formed during CO_2_ reduction, aliquots (1 mL) of the electrolyte solution were collected at different time intervals. The collected aliquots were diluted, neutralized, and analyzed by ^1^H and ^13^C NMR spectroscopy using dimethyl sulfoxide (DMSO) as an internal standard. The NMR peak area of DMSO (at 2.6 ppm) was set at 1.0 and used to calculate the NMR peak area of formate (at 8.35 ppm). The number of moles of formate was calculated by using the formula, *n* = 0.03*L* × formate concentration (M), where *L* is the volume of the electrolyte and *n* (=2) is the number of electrons involved in the formation of formate. Then subsequently FE was calculated using the following equation,
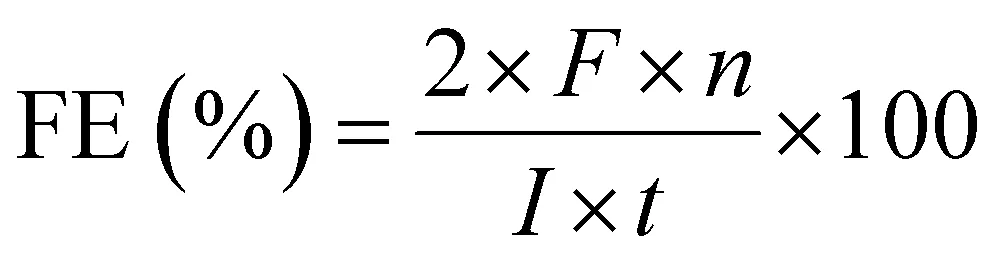
where *F* is Faraday's constant (96 500 C mol^−1^), *I* is the current, and *t* is the time duration. Gaseous products were determined using a micro gas chromatograph (GC) (Agilent MicroGC 990). Gas samples were collected from the headspace of the cell and analyzed by the GC.

### Theoretical studies

Unrestricted density functional theory (DFT) was employed to optimize the model complex and the complex with Rbf. The B3LYP functional and def2-TZVP basis set were used for the calculations. The Grimme D3-BJ dispersion correction scheme was applied to account for nonbonding interactions. All the calculations were performed using the Turbomole v7.5 package.

## Results and discussion

Cu-MOF was synthesized and subsequently modified with Rbf through a simple mechanochemical method, as described in the experimental section (Fig. S1). Both Cu-MOF and Rbf–Cu-MOF were characterized using different physicochemical characterization techniques to determine the effects of PSM of Cu-MOF with Rbf. The morphology of the samples was studied by SEM and HR-TEM. The SEM images of Cu-MOF showed a flake-like structure (Fig. 1a) which is consistent with the morphology reported for Cu-4,4′-bpy-based MOFs under similar synthetic conditions.^25,26^ Further, HR-TEM reveals a two-dimensional sheet type morphology (Fig. 1b) for Cu-MOF. The high-resolution lattice fringes and diffraction spots in the selected area electron diffraction (SAED) pattern indicate a highly ordered crystalline nature of Cu-MOF (Fig. 1c and inset). Upon PSM with Rbf, smaller particles were observed, as shown in Fig. 1d and e, which corresponded to Rbf particles, while the underlying Cu-MOF is retained (additional data are provided in the SI; Fig. S2 and S3). Further, HR-TEM depicts discontinued lattice fringes and disordered structure, and the corresponding SAED pattern shows diffused rings with less spots, confirming the transition to a slightly amorphous structure. STEM-HAADF coupled with elemental mapping analysis of Rbf–Cu-MOF showed a uniform distribution of C, O, Cu and N throughout the sample (Fig. 2a–f).^27^

**Fig. 1:**
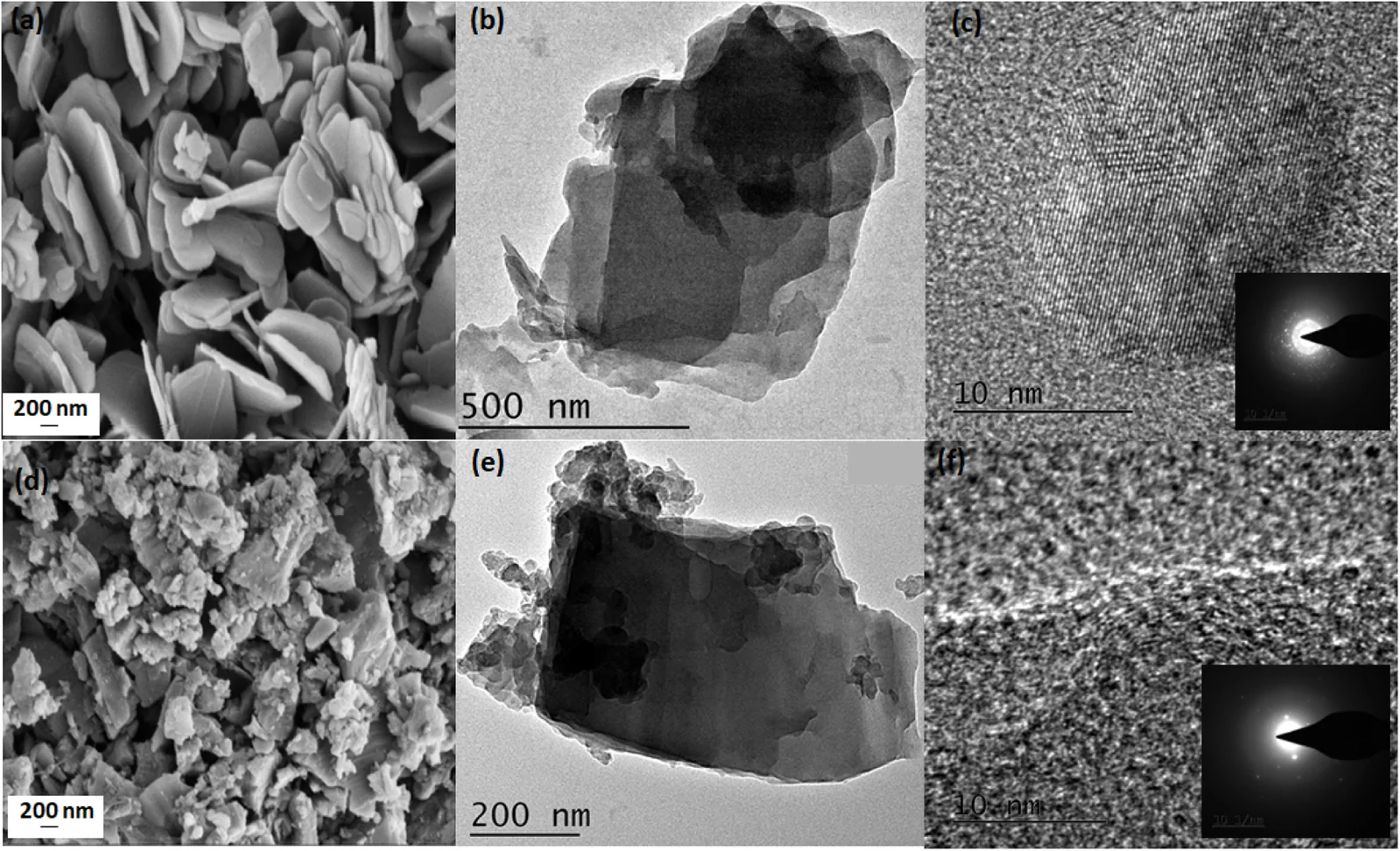
(a) SEM and (b and c) TEM images of Cu-MOF, and (d) SEM and (e and f) TEM images of Rbf–Cu-MOF.

**Fig. 2:**
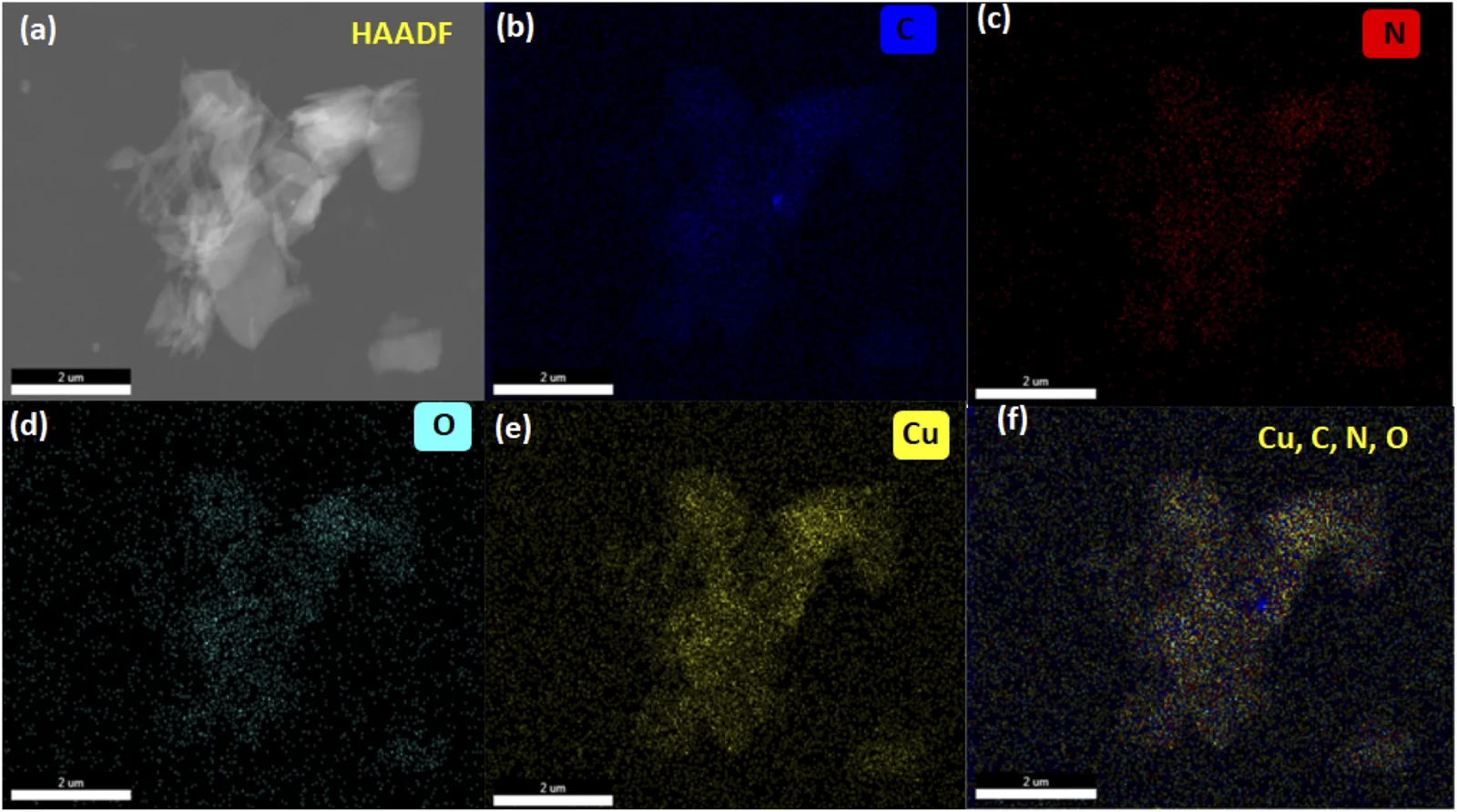
(a) HAADF-STEM image of Rbf–Cu-MOF, and (b–f) elemental mapping of various elements present in Rbf–Cu-MOF.

Single crystal X-ray diffraction (SXRD) revealed that the Cu-MOF crystallized in the monoclinic space group *P*12_1_/*c* 1 with unit cell dimensions: *a* = 18.6865 Å, *b* = 11.9234 Å, *c* = 7.3841 Å, *α* = *γ* = 90°, and *β* = 96.971° (Fig. 3a, S4–S8, and Table S1). The copper center was coordinated by two nitrogen atoms from 4,4′-bpy (Cu–N ≈ 2.00 Å) and two oxygen atoms from water molecules (Cu–O ≈ 1.90 Å), forming a robust N_2_O_2_ inner coordination environment. Additionally, two longer Cu⋯O interactions (∼2.37 Å) from nearby nitrate ions were observed axially (Fig. S4). Although the six proximate donor atoms might suggest an octahedral geometry, the significant disparity in bond lengths, particularly the elongated Cu–O(NO_3_) and supporting hydrogen-bonding interactions (H⋯O_NO_3_ = 2.64–3.26 Å) indicated that these nitrate ligands were not strongly coordinated. Instead, they resided in the outer coordination sphere and were stabilized by hydrogen bonding with aqua ligands. These geometric and interaction features supported the assignment of a pseudo-octahedral geometry in which the Cu(i) center is formally four-coordinated (N_2_O_2_) with two weakly associated nitrates aligned axially.

This interpretation was reinforced by X-ray absorption fine structure (XAFS) data which showed a measurable change in the Cu–O(NO_3_) scattering shell before and after electrochemical treatment. The partial loss or evaporation of the Cu⋯NO_3_ interactions observed post-electrolysis confirmed the lability of the nitrate ligands and supported their non-covalent association in the pristine structure. In contrast, Cu–N and Cu–O(water) shells remained unchanged, affirming their role as inner-sphere ligands. The elongation of axial bond looked like a Jahn–Teller-elongated octahedron, with the axial Cu–O(NO_3_) bonds greatly elongated. Here, the elongation arose not from Jahn–Teller distortion [Cu(i) has no such distortion since its d-shell is full] but simply from the weakness of Cu⋯NO_3_ interaction. The absence of any Jahn-Teller distortion in the structure [as confirmed by the equal short Cu–O(H_2_O) distances and the spectroscopic identification of Cu(i)] was fully consistent with the d^10^ Cu(i) center. All evidences (XPS binding energy ∼932.2 eV, typical of Cu^+^, and XAFS analysis) confirmed that the metal was Cu(i); therefore, the elongated geometry must be attributed to chemical bonding factors (ligand strength and geometry) rather than electronic degeneracy.

The observed Cu–O(NO_3_) distances (∼2.37 Å) fell at the boundary between true coordination and long-range electrostatic or hydrogen bonding interactions. The literature precedent for Cu(i)–NO_3_ complexes consistently showed that when stronger ligands were available, nitrate typically adopted an outer-sphere position or weakly coordinated *via* bridging or monodentate fashion, often with Cu–O distances ≥2.3 Å.^26^ The present structure closely matched this behavior.

Finally, PXRD analysis of Cu-MOF (Fig. 3b) confirmed that the bulk material matched the SXRD-resolved structure, confirming phase purity. Rbf–Cu-MOF displayed diffraction features identical to those of Cu-MOF, indicating that PSM with Rbf did not disrupt the crystalline integrity. Strong reflections in the 2*θ* = 10°–20° region are characteristic of long-range order and microporosity in the 4,4′-bpy-based MOFs, consistent with previous reports.^25^

**Fig. 3:**
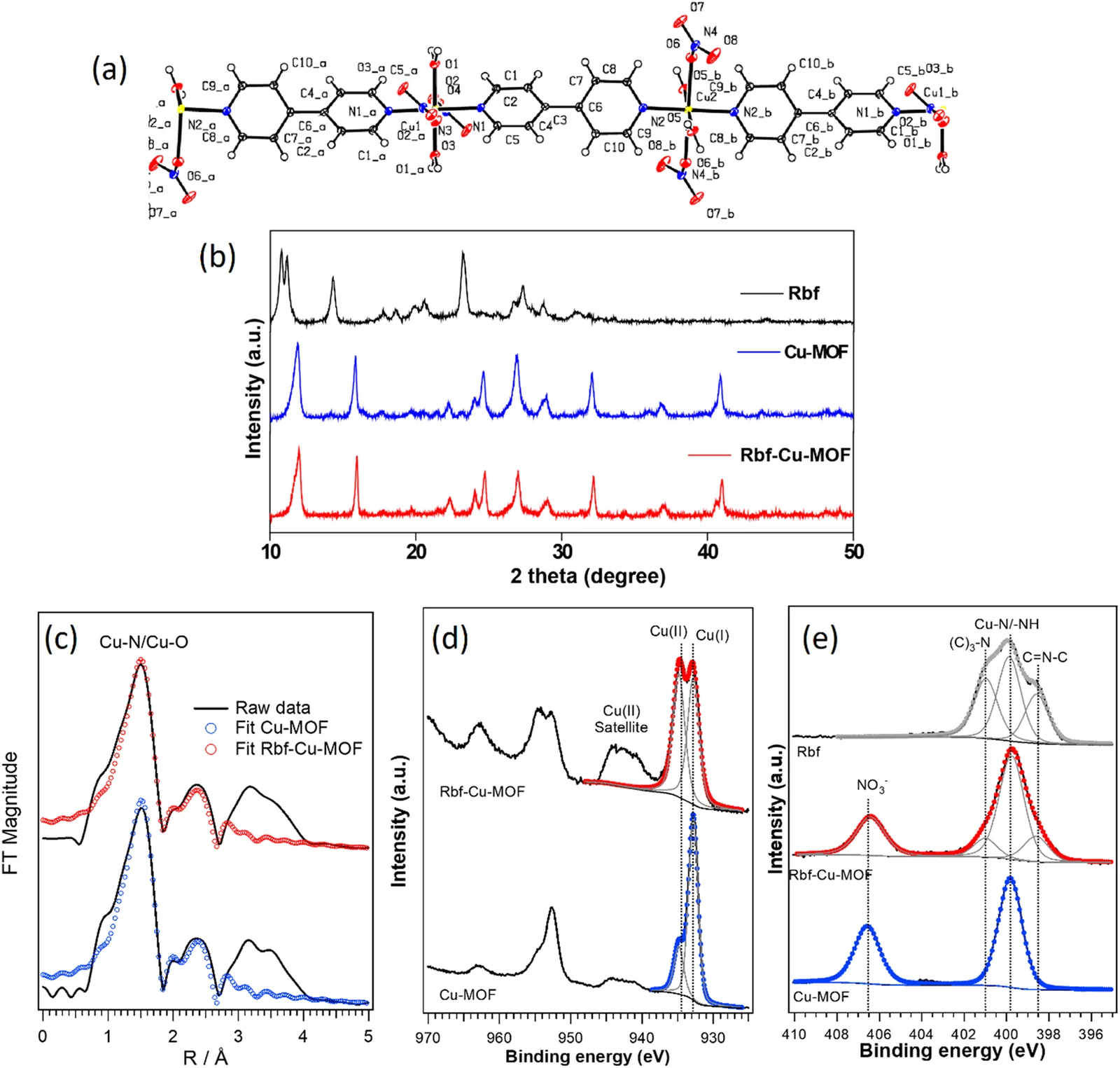
(a) Crystal structure of Cu-MOF, (b) PXRD patterns, (c) phase-uncorrected FT-EXAFS at the Cu K edge, with the corresponding fit, (d) Cu 2p and (e) N 1s XPS regions of pristine Cu-MOF and Rbf–Cu-MOF. The PXRD pattern and N 1s spectrum of Rbf are included in (b) and (e), respectively, for comparison.

The structure and coordination of the Cu ions were investigated using X-ray absorption spectroscopy (XAS). Analysis of the X-ray absorption near-edge structure (XANES) data indicated that the Cu ions had an oxidation state close to +1 (∼+1.15) in both the materials (Fig. S9), suggesting that the addition of Rbf did not affect the Cu oxidation state. The corresponding phase-uncorrected Fourier transform-extended X-ray absorption fine structure (FT-EXAFS) for both the materials, reported in Fig. 3c, were very similar. They showed an intense peak at 1.55 Å, which could be related to Cu coordinated to N and/or O atom(s),^28^ while additional peaks at *R* > 2.3 Å could be attributed to carbon atoms in the MOF structure. No peaks attributed to Cu–Cu interactions were observed at 2.2 Å; therefore, the decomposition and/or agglomeration of Cu atoms during the MOF synthesis or mechanochemical treatment could be excluded. The crystal structure of the MOF, as shown in Fig. 3a, was confirmed by fitting the EXAFS data (Table S2). In both the samples, Cu ions had two oxygen neighbor atoms at 1.94 Å from water molecules, two nitrogen neighbor atoms at 2.04 Å from the 4,4′-bpy units, two oxygen atoms at 2.63 Å from nitrate molecules, and four carbon atoms at 2.92 Å. This suggested that Rbf did not modify the Cu-MOF structure as expected, since Rbf particles were deposited on the surface of Cu-MOF and not inserted into its structure, as observed in the SEM and TEM images (Fig. 1). Therefore, if there was any interaction between the Rbf and Cu-MOF units, it should have occurred on the surface and not in the bulk.

XPS measurements were performed to explore the possible interactions between the two units. Fig. 3d shows the Cu 2p XPS region for the two Cu-MOF-based samples. The fit of the Cu 2p_3/2_ region for the pristine Cu-MOF indicated that Cu was present in different oxidation states. The first component, centred at 932.8 eV, could be associated with the presence of Cu(i), in agreement with the XANES analysis. In contrast, the second component at 934.5 eV in the Cu 2p_3/2_ peak, as well as the intense satellite peak at 942.0 eV, were related to the presence of Cu(ii) on the surface of the MOF.^29,30^ The small amount of Cu(ii) (29.3%) could be attributed to the oxidation of Cu(i) in contact with air. After the addition of Rbf, the same two components were observed in the Cu 2p_3/2_ region. However, an increase in the Cu(ii) component (66.8%) was observed with respect to Cu(i), which could be related to the interaction of Cu ions with Rbf units. The N 1s region was also analyzed to gain insights into the Rbf and Cu-MOF interactions (Fig. 3e). Cu-MOF presented only two components at 399.8 and 406.6 eV related to Cu–N and NO_3_^−^ species, respectively.^31^ Rbf, on the other hand, presented three components at 398.6, 399.9 and 401.0 eV, attributed to C=N–C, NH, and C_3_–N, respectively.^32^ Analysis of N 1s region of Rbf–Cu-MOF showed the presence of N from both the Cu-MOF and Rbf. The main components were observed at 399.8 and 406.6 eV, associated with Cu–N and NO_3_^−^ species, respectively, from Cu-MOF. In addition, smaller components at 398.6 and 401.0 eV could be attributed to C=N–C and C_3_–N, respectively, from Rbf. The NH component of Rbf overlapped with the Cu–N of Cu-MOF. Additional characterization by FT-IR spectroscopy, UV-vis spectroscopy, and N_2_-physisorption, analysis was performed to determine the chemical, optical, and textural properties of Rbf–Cu-MOF. The details can be found in the SI (Fig. S10–S14).

### Electrochemical characterization

The electrocatalytic performances of Cu-MOF and Rbf–Cu-MOF were assessed *via* CV and LSV techniques in N_2_- and CO_2_-saturated 0.5 M NaHCO_3_ electrolytes. As seen in Fig. 4a and b, the multiple redox features observed for Cu-MOF and Rbf–Cu-MOF could be due to the overlapping redox and interfacial processes. For Cu-MOF, the cathodic peaks were primarily associated with potential dependent reduction of Cu species and connected with electrochemical restructuring of the Cu coordination environments. Such potential dependent surface reconstruction was well documented for Cu based CO_2_ reduction catalysts under cathodic polarization.^33^ In Rbf–Cu-MOF, the redox peaks observed at −0.3 and −1.3 V (*vs.* RHE) were attributed to the intrinsically redox-active isoalloxazine moiety of Rbf molecules, which could undergo sequential one-and two-electron transfer processes.^35^ Therefore, the multiple features observed for Rbf–Cu-MOF are mainly associated with the combined contributions of Cu-centered restructuring events and redox shuttles of Rbf molecules. To further understand the electrochemical responses, multiple CV cycles were performed under CO_2_- and N_2_-saturated conditions. As shown in Fig. S16a–d, the CVs exhibited noticeable changes in the initial cycles followed by relatively stable response, suggesting the gradual formation and stabilization of the electrochemical interface between the electrode and electrolyte. Further, in the absence of CO_2_, reduction in current densities was observed at more negative than −1.3 V (*vs.* RHE), which could be attributed to the hydrogen evolution reaction (HER). In the presence of CO_2_, an increase in current density and a shift in the onset potential towards less negative potential was observed, suggesting that CO_2_ was reduced. By comparing the results obtained for Cu-MOF and Rbf–Cu-MOF, it was clear that the presence of Rbf significantly increased the catalytic activity towards the CO_2_ reduction reaction (CO_2_RR) (Fig. S15a). It was well documented that the presence of NH_2_ group in the MOFs improved the CO_2_ uptake.^33,34^ In the Rbf–Cu-MOF system, the presence of NH groups and N atoms (from Rbf) close to the Cu centers favored the adsorption of CO_2_ and, thereby improving the CO_2_RR activity.^34^ EIS measurements were performed to investigate the electron transfer rate across the electrode–electrolyte interface. Fig. S15b shows that Rbf–Cu-MOF had a smaller ESR (2.5 Ω) than the pristine Cu-MOF (3.0 Ω), which reflected the better electron transfer kinetics in Rbf–Cu-MOF owing to the decreased charge-transfer resistance. The presence of Rbf facilitated charge transfer across the electrode–electrolyte interface, thereby enhancing the electrocatalytic activity.^36–39^

**Fig. 4:**
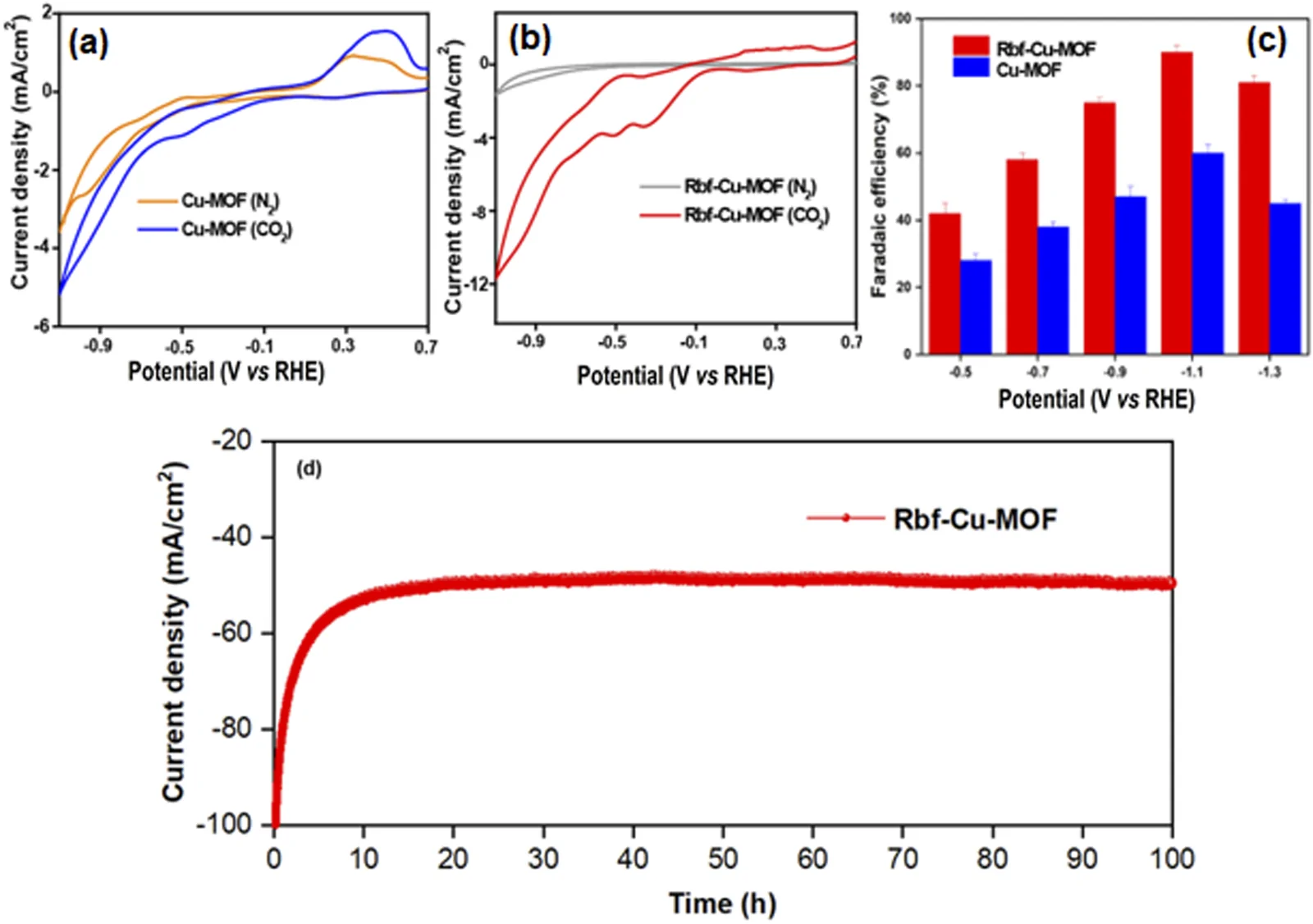
(a and b) CVs recorded in N_2_- and CO_2_-saturated 0.5 M NaHCO_3_ at a sweep rate of 50 mV s^−1^ on Cu-MOF and Rbf–Cu-MOF (1 : 1), (c) faradaic efficiency determined at various potentials for Cu-MOF and Rbf–Cu-MOF (1 : 1), and (d) chronoamperometry at −1.1 V *vs.* RHE of Rbf–Cu-MOF (1 : 1).

To investigate the concentration and role of Rbf, three samples with different Cu-MOF : Rbf molar ratio (1 : 0.5, 1 : 1, 1 : 2) were prepared and tested as CO_2_RR catalysts. As shown in Fig. S17, the addition of Rbf increased the catalytic performance of Cu-MOF in all the cases. An increase in current density was observed with the amount of Rbf for samples 1 : 0.5 and 1 : 1. However, a further increase in the amount of Rbf had a negative effect on the performance, probably due to the lower number of active sites (Cu ions) or filling of the pores with the consequent blocking of the active Cu centres by Rbf molecules. Therefore, 1 : 1 Rbf–Cu-MOF showed the best catalytic performance in terms of current density (15 mA cm^−2^ at −1.1 V *vs.* RHE) and onset potential (−0.1 V *vs.* RHE) (Fig. S17). Hence, further studies on the CO_2_RR products were performed using this sample.

Long-term electrolysis experiments were performed at different potentials to determine the product distribution as a function of the applied potential. The current density profile of Rbf–Cu-MOF (1 : 1) at the selected potentials is shown in Fig. S18. Upon varying the potential from −0.3 to −1.3 V (*vs.* RHE), a steady increment in the current density was observed for Rbf–Cu-MOF (1 : 1), and it was stable for at least 100 min. To determine the CO_2_RR products as a function of the applied potential, aliquots of the electrolyte (1 mL) from the cathode chamber were collected at different time intervals and analyzed by ^1^H- and ^13^C-NMR. Notably, both Cu-MOF and Rbf–Cu-MOF successfully converted CO_2_ into HCOO^−^, as confirmed by NMR analyses. Upon careful evaluation of the NMR spectra, it was noted that at −0.5 and −0.7 V (*vs.* RHE), there were no peaks corresponding to HCOO^−^ in the case of pristine Cu-MOF (Fig. S19). Interestingly, at −0.5 V (*vs.* RHE), Rbf–Cu-MOF (1 : 1) displayed small peaks at chemical shift values of 8.35 and 160 ppm in ^1^H- and ^13^C-NMR spectra, respectively, which confirmed the formation of HCOO^−^. While comparing the catalytic activities of Cu-MOF and Rbf–Cu-MOF (1 : 1), it was clear that Rbf–Cu-MOF (1 : 1) started to convert CO_2_ at a less negative potential −0.5 V (*vs.* RHE) (Fig. S20). At more negative potentials (−0.7, −0.9, and −1.1 V *vs.* RHE), an increment in the HCOO^−^ peak intensity was noticed. The yield of HCOO^−^ reached a maximum at a potential of −1.1 V (*vs.* RHE), and a further rise in the potential had no effect on the HCOO^−^ formation. Instead, it favored competitive HER. Hence, further studies were conducted at −1.1 V (*vs.* RHE). The conversion of CO_2_ to HCOO^−^ and the corresponding FE were determined using the Faraday equation, as mentioned in the experimental section. The relative intensity of the signals related to HCOO^−^ increased sharply with time. Cu-MOF showed a FE of 59% towards HCOO^−^ production with a current density of −9 mA cm^−2^. At the same potential (−1.1 V *vs.* RHE), Rbf–Cu-MOF (1 : 1) displayed a higher current density (−22 mA cm^−2^) with a maximum FE of 90% towards HCOO^−^ (Fig. 4c). The enhancement in the quantity of HCOO^−^ and FE might be linked to the inherent electrical characteristics of Rbf–Cu-MOF. To check the long-term durability and quantify the product at different time intervals, CO_2_ electrolysis was continued for 100 h (Fig. 4d). The products were identified using NMR spectroscopy with DMSO as an internal standard (Fig. S22). It was confirmed that HCOO^−^ was the primary product, along with a meagre quantity of methanol. To identify the gaseous products, GC analysis was performed by collecting the gas from the headspace of the electrochemical cell. The corresponding chromatograms are presented in Fig. S23 and S24, which showed the formation of small quantities of H_2_ and CO along with the traces of ethylene, ethane, and methane.

### Stability under CO_2_RR conditions

To investigate the chemical and morphological stability of Cu-MOF under CO_2_RR conditions as well as the role of Rbf, both the samples were analyzed after 10 h of electrolysis (Fig. 4d and S25) at −1.1 V (*vs.* RHE) by HR-TEM and XAS. Fig. 5a–c displays the HR-TEM and STEM images of Cu-MOF. It could be observed that the post-electrolysis Cu-MOF particles had a different shape compared to the original ones (Fig. 1b), suggesting the partial destruction of the MOF structure. In addition, large metal agglomerates were observed, demonstrating that the Cu ions were unstable under the catalytic conditions. The reduction of Cu(i) to metal Cu and the consequent formation of nanoparticles have been already reported for similar materials.^19,20,37^ On the other hand, post-electrolysis images of Rbf–Cu-MOF did not show any significant difference compared to the fresh one (Fig. 5d–f). Cu nanoparticles were not observed in this case, suggesting that Rbf could stabilize the Cu-MOF. In addition, post SEM-EDX (Fig. S26a–d) and XPS (Fig. S27 and S28) analyses of the spent electrodes showed the well-preserved sheet like morphology, which further confirmed the stability of Rbf–Cu-MOF. Rbf has a chelation tendency due to the presence of OH and NH groups. It could form chelate with the coordinatively unsaturated Cu-MOF and stabilize it, resulting in enhanced catalytic activity and stability (Fig. 4d).^38,40,41^

**Fig. 5:**
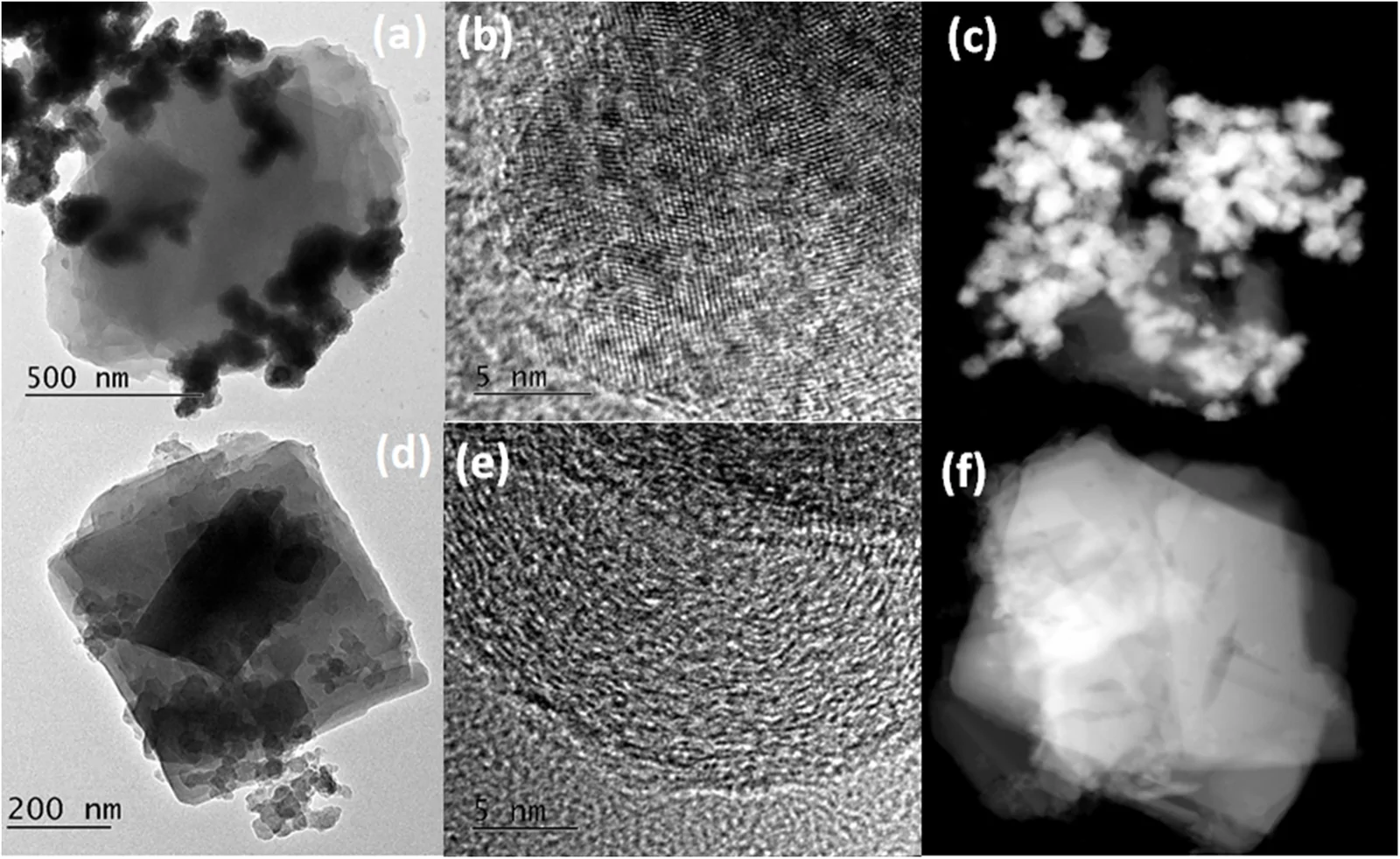
Post-electrolysis HR-TEM and STEM images of (a–c) Cu-MOF, and (d–f) Rbf–Cu-MOF.

Fig. 6a shows the XANES spectra of the samples before and after electrolysis.^42,43^ For Cu-MOF, a pre-peak at 8981.2 eV could be attributed to the reduction of Cu(i) to Cu(0), and the formation of Cu clusters/nanoparticles started to grow after electrolysis, in agreement with the Cu nanoparticles observed by HR-TEM. Their presence could be explained by the migration of Cu atoms under CO_2_RR conditions and their consequent agglomeration, as previously reported in the literature.^38,44–47^ In contrast, XANES spectra of the fresh and post-electrolysis samples of Rbf–Cu-MOF were very similar, confirming the higher stability of the Cu ions, which could be related to the presence of Rbf. Fig. 5b shows the FT-EXAFS for both the samples and their comparison with the fresh samples. For Cu-MOF, a clear change was observed in the plot. An increase in the intensity at 2.2 Å after electrolysis suggested the existence of Cu⋯Cu interactions, in agreement with the formation of Cu clusters. In addition, the decrease of the peak at 1.55 Å suggested the decrease of the Cu⋯N and Cu⋯O interactions, also in agreement with the formation of Cu clusters. In the case of Rbf–Cu-MOF, the shape of the two curves was the same, just a slight decrease in the intensity of the peaks at *R* > 2.3 Å was observed, which could indicate a partial destruction of the organic part of the MOF. It was possible to fit the data for Rbf–Cu-MOF, and the fitting parameters reported in Table S3 confirmed the stability of the material after electrolysis. In contrast, it was not possible to fit the data for Cu-MOF; because, it was formed by two different Cu phases, Cu-based clusters, and Cu-MOF, as demonstrated by HR-TEM.^48^

**Fig. 6:**
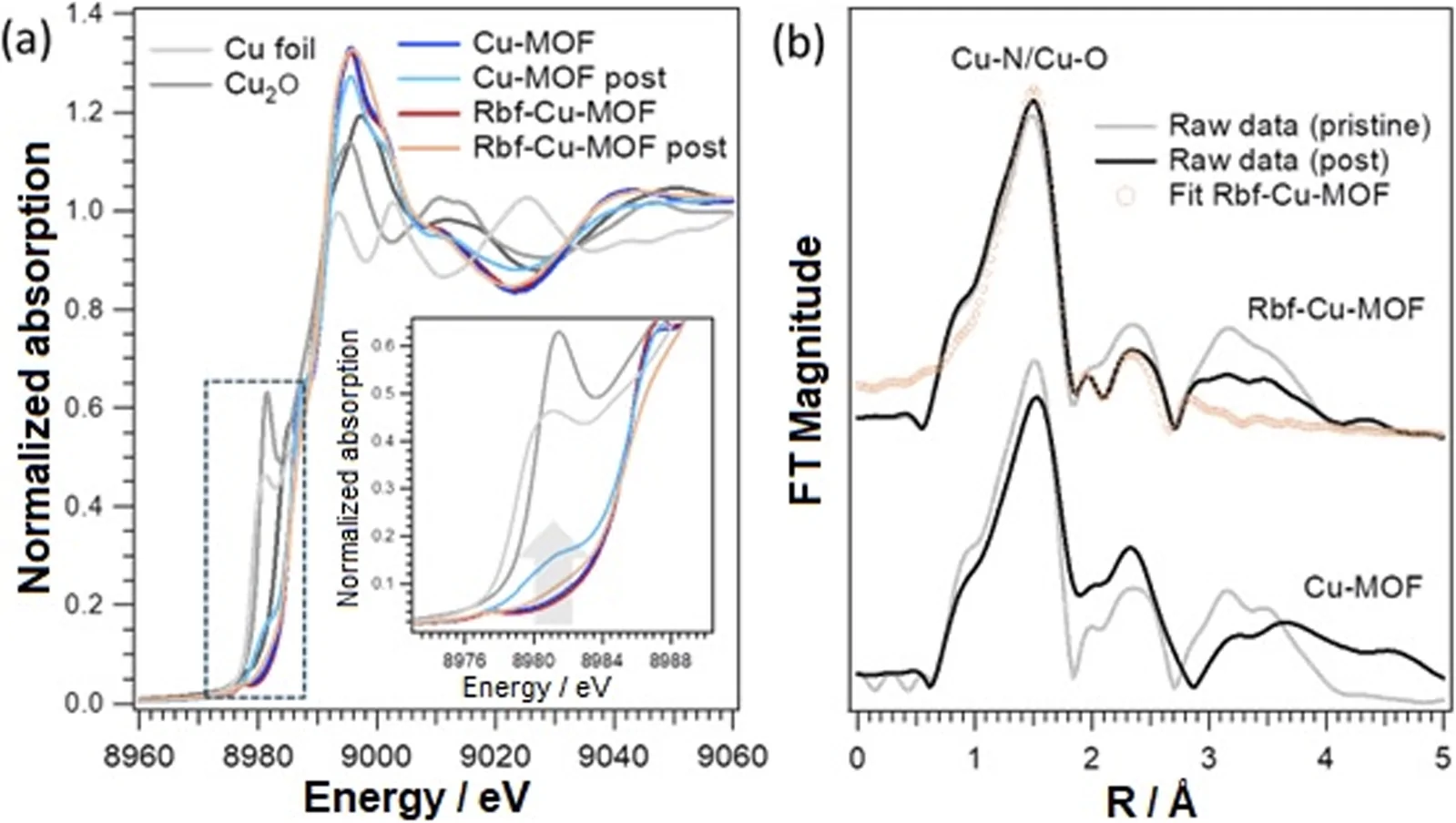
(a) XANES region, and (b) phase-uncorrected FT-EXAFS spectra at the Cu K edge and corresponding fits for Cu-MOF and Rbf–Cu-MOF after electrolysis. The inset in (a) is a magnification of the dotted rectangle showing the pre-edge peak.

### Theoretical studies

DFT calculations of Cu-MOF and Rbf–Cu-MOF were performed using a single molecule structure of Cu-MOF obtained from the crystal structure depicted in Fig. 3a, and the systems were optimized using the spin-polarized DFT method. The Rbf–Cu-MOF structure was optimized using one molecule of Rbf attached to the Cu center of Cu-MOF, and the electronic interaction between Cu-MOF and Rbf was investigated. The computational studies have provided the basic electronic structures of these systems. The energy calculations revealed that the spin-orbital distributions of Cu-MOF and Rbf–Cu-MOF differed significantly. The addition of Rbf to the pristine MOF altered the electronic structure of the Cu-MOF. For pristine Cu-MOF, HOMO and HOMO − 1 (singly occupied MO with *α*-spin) were localized on the nitrate ligands. The LUMO(*β*) of pristine Cu-MOF was localized on one Cu and LUMO + 1(*β*) on another Cu. Therefore, the electronic structure of pristine Cu-MOF showed that the nitrate ligands could facilitate the electron transfer for CO_2_ reduction. In the case of Rbf–Cu-MOF, HOMO(*α*) and HOMO − 1(*β*) were localized on the Rbf molecule, and the energy levels were shifted to approximately 0.5 eV higher than that of the pristine HOMO. In contrast, LUMO(*β*) and LUMO + 1(*β*) were localized on Cu for both Cu-MOF and Rbf–Cu-MOF. Interestingly, the LUMO(*β*) of Rbf–Cu-MOF was reduced by approximately 0.3 eV compared to the LUMO(*β*) of Cu-MOF. The increase in the HOMO energy and decrease in the LUMO energy reduced the HOMO–LUMO gap of Rbf–Cu-MOF compared to that of pristine Cu-MOF, which could enhance the catalytic activity. Since the HOMO(*α*) and HOMO − 1(*β*) were localized on Rbf molecule in Rbf–Cu-MOF, Rbf modified the electronic structure and facilitated charge transfer. This improved the catalytic performance of Rbf–Cu-MOF compared to that of pristine Cu-MOF, which was consistent with the experimental results (Fig. 4). Moreover, the addition of Rbf resulted in a different Cu–N and Cu–O bond lengths (Fig. S5–S8). Here, the Cu–N was nothing but Cu–4,4′-bpy connection, which was reduced by 0.03 Å, and Cu–O was Cu–water connection, which was reduced by around 0.2 Å. The optimized structures and coordinates of Cu-MOF and Rbf–Cu-MOF are shown in Fig. 7 and 8, respectively.

**Fig. 7:**
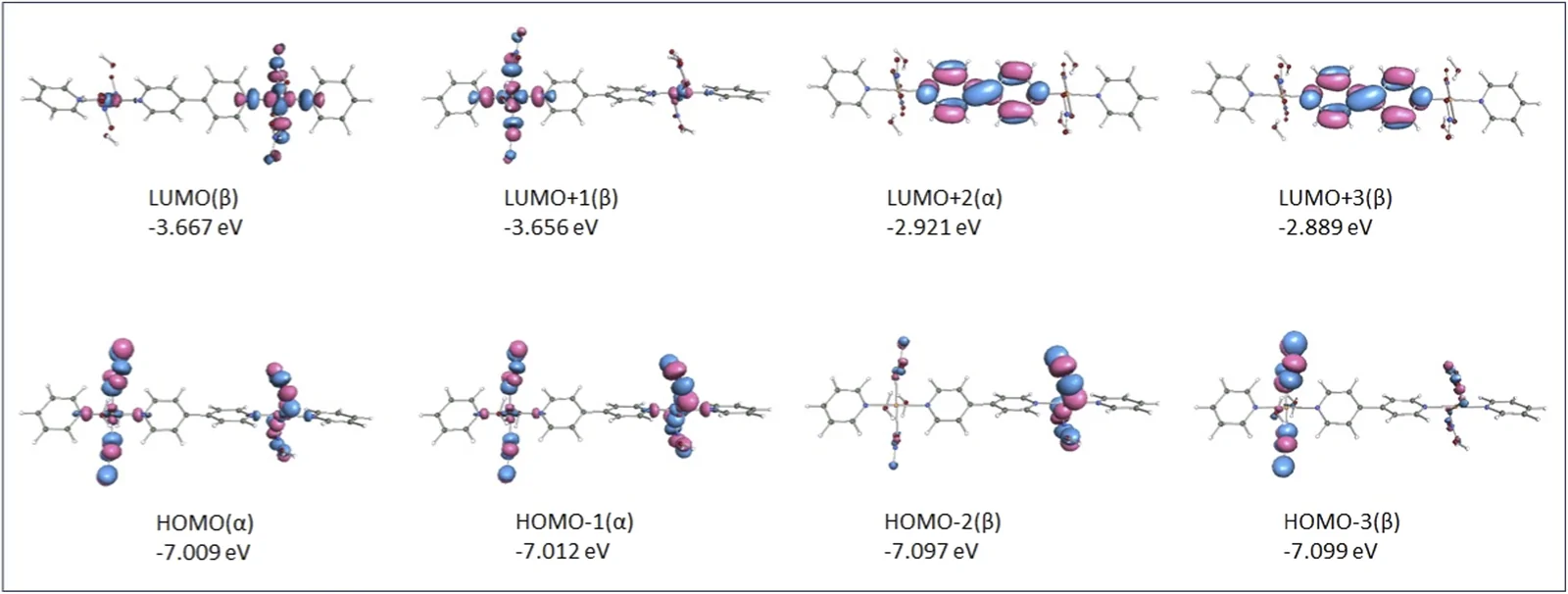
Frontier spin-orbitals of Cu-MOF.

**Fig. 8:**
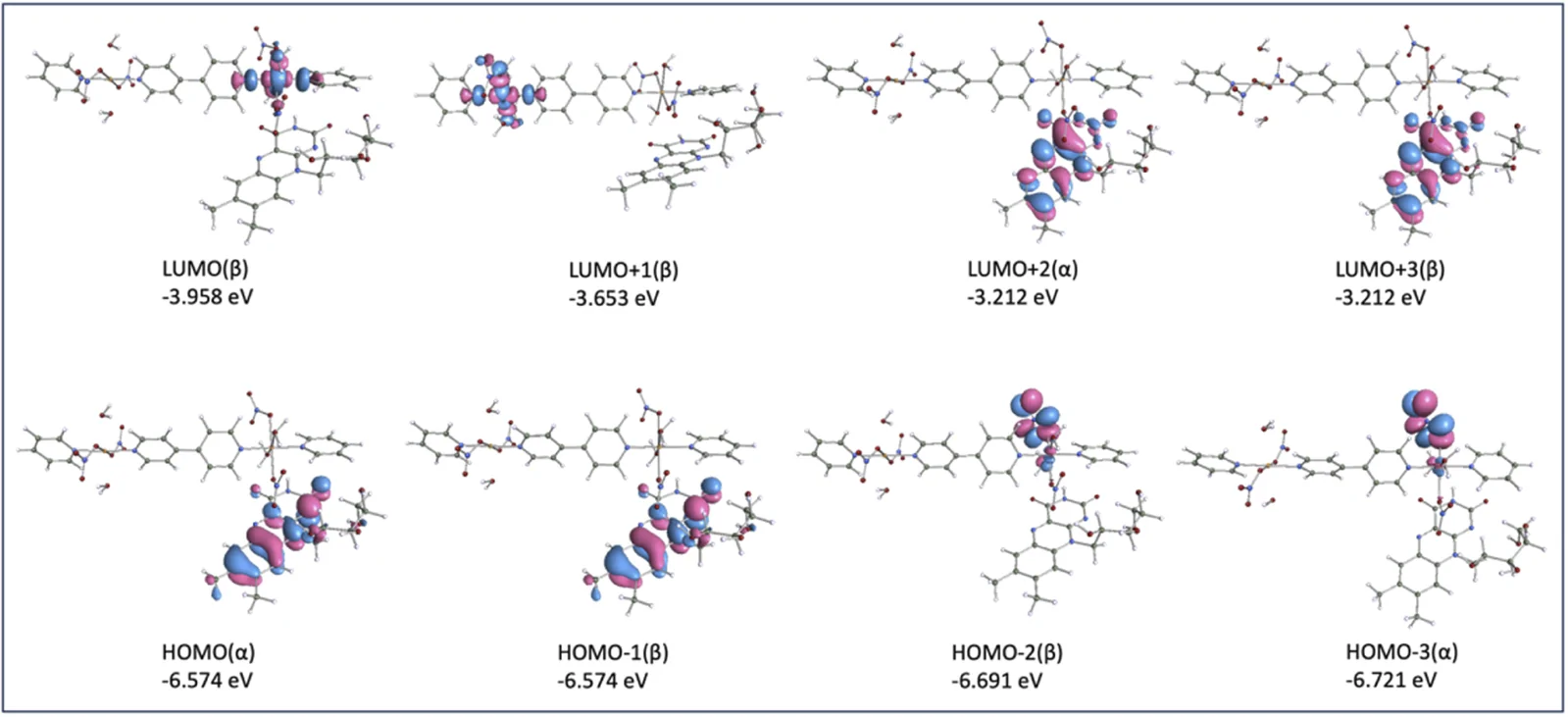
Frontier spin-orbitals of Rbf–Cu-MOF.

## Conclusions

A Cu-MOF with a sheet-like morphology was prepared using water as a solvent under low-temperature condition. The Cu-MOF was then modified with Rbf through a simple and facile mechanochemical method; however, the presence of Rbf did not modify the chemical and structural properties of Cu-MOF. Rbf–Cu-MOF was tested as a catalyst for the electrocatalytic reduction of CO_2_. Both Cu-MOF and Rbf–Cu-MOF showed significant CO_2_ conversion to form formate. Interestingly, Rbf–Cu-MOF (1 : 1) showed three-fold higher electrocatalytic activity than pristine Cu-MOF, which could be attributed to the electrons supplied by Rbf, as shown by DFT calculations. In addition, Rbf helped to stabilize the Cu ions, avoiding their migration and agglomeration with the consequent and irreversible formation of Cu nanoparticles, as occurred in its absence. Therefore, it could be concluded that the presence of Rbf contributes to (i) favoring CO_2_ uptake, enhancing CO_2_ reduction, and suppressing HER activity; (ii) stabilizing Cu ions in the MOF structure under electrolysis conditions through a chelation effect, avoiding the formation of Cu nanoparticles; and (iii) supplying electrons to the CO_2_ reduction reaction, enhancing the catalytic activity of Cu-MOF. Furthermore, by replacing hazardous solvents with water and mechanical energy, and utilizing a bio-derived modifier that ensures high operational stability, the Rbf–Cu-MOF system presents a significantly lower “cradle-to-gate” carbon footprint than conventional MOF-based electrocatalysts.

## Conflicts of interest

The authors declare no competing financial interest.

## Supplementary Material

RA-OLF-D6RA05541A-s001

## Data Availability

Data will be made available upon request. All data supporting the findings of this study are given in the article and its supplementary information (SI). Supplementary information is available. See DOI: https://doi.org/10.1039/d6ra05541a.
