# Supplementary material for: Post synthetic modification of Cu-MOF with riboflavin for stable electrocatalytic conversion of CO2 to formate
Source: RSC Adv. 2026 Nov 10. Online ahead of print. doi: 10.1039/d6ra05541a (PMC13749341; doi:10.1039/d6ra05541a)
Supplement: Supplementary file 1 [file RA-OLF-D6RA05541A-s001.pdf]

Supplementary Information (SI) for RSC Advances.
This journal is © The Royal Society of Chemistry

Supplementary Information

for

Post Synthetic Modification of Cu-MOF with Riboflavin for Stable Electrocatalytic Conversion of CO_2 to Formate

Pandiaraj Sekar,[a,e] Punitharaj Vasanthakumar,[a] Selvaraj Paramasivam,[b] Rajasekharan Jayakumari Deepak,[a] Laura Calvillo,*,[c] Sunandan Sarkar,[a] Nattamai Bhuvanesh,[d] Kasthuri Annamalai Sami,[b] Shanmugam Senthil Kumar*,[b] and Ramasamy Karvembu*,[a]

[a] Department of Chemistry, National Institute of Technology, Tiruchirappalli 620015, India

[b] Electrodics and Electrocatalysis Division, CSIR-Central Electrochemical Research Institute, Karaikudi 630006, India

[c] Department of Chemical Sciences, University of Padova, Via F. Marzolo 1, 35131 Padova, Italy

[d] Department of Chemistry, Texas A & M University, College Station, Texas 77842, United States

[e] Department of Physics, American University of Sharjah, Sharjah 26666, United Arab Emirates

Corresponding authors: R. Karvembu, E-mail: kar@nitt.edu; S. S. Kumar, E-mail: ssenthilkumar@cecri.res.in; and L. Calvillo, E-mail: laura.calvillolamana@unipd.it

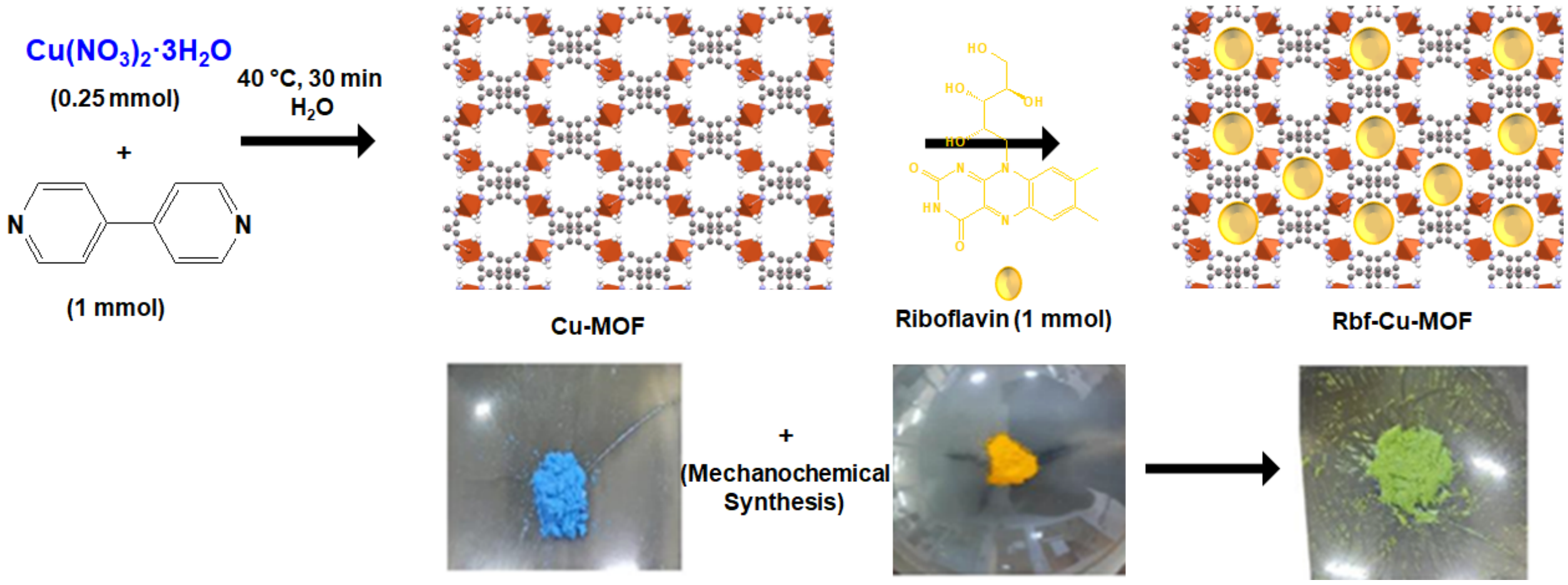

Fig. S1. Schematic illustration of the synthesis of Cu-MOF and introduction of Rbf onto Cu-MOF. Digital images represent the colour change of the MOF during the mechanochemical synthesis of Rbf-Cu-MOF.

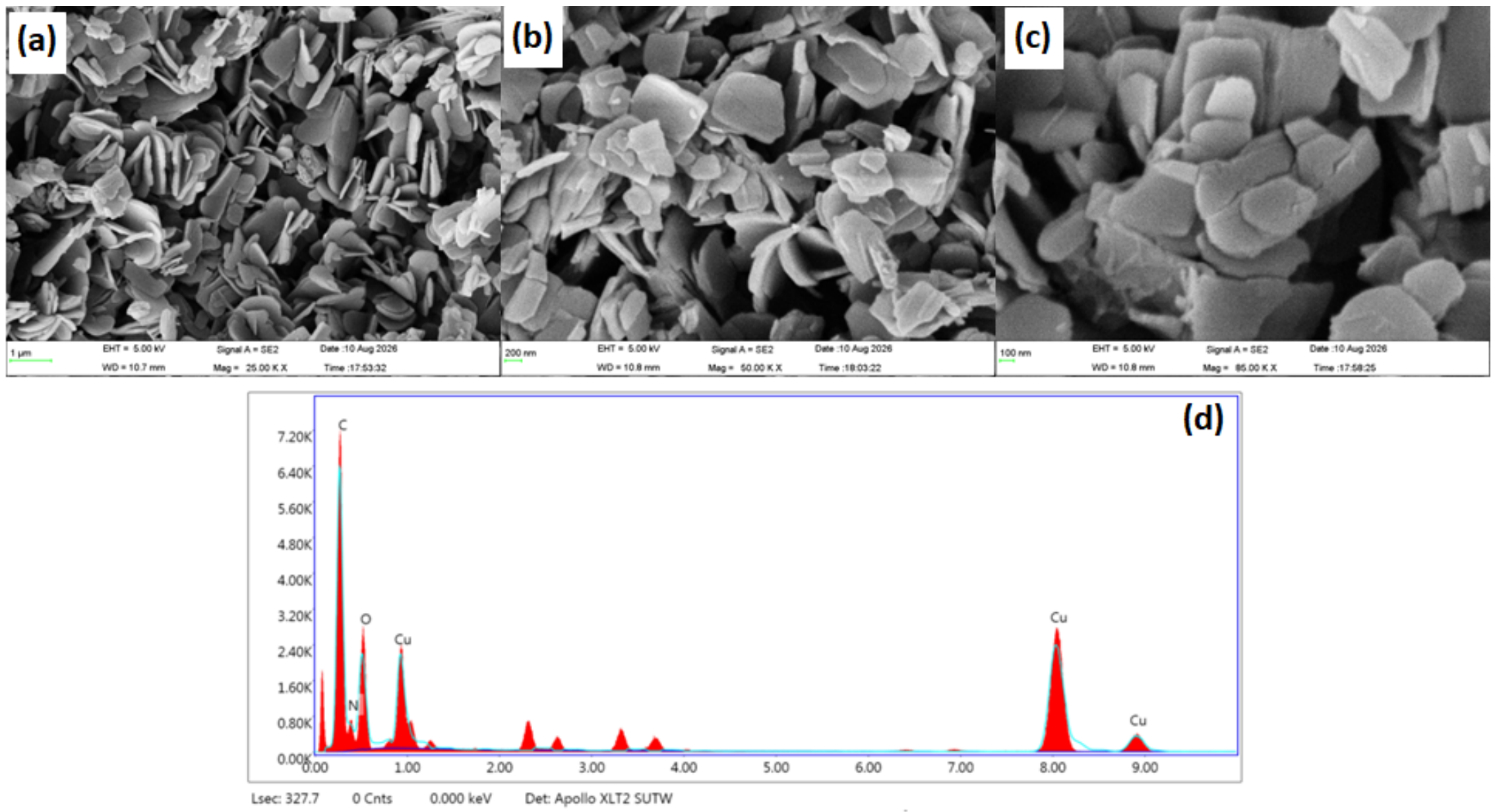

Element	Weight %	Atomic %	Net Int.	Net Error%	KABFactor
C K	38.31	64.63	351.90	0.74	1
N K	2.00	2.90	11.30	22.13	1.63
O K	14.18	17.95	109.90	2.54	1.18
CuK	45.51	14.51	301.90	0.95	1.38

Fig. S2. (a, b and c) SEM images of Cu-MOF at different magnifications, and (d) EDX spectrum and table show the composition of various elements present in Cu-MOF.

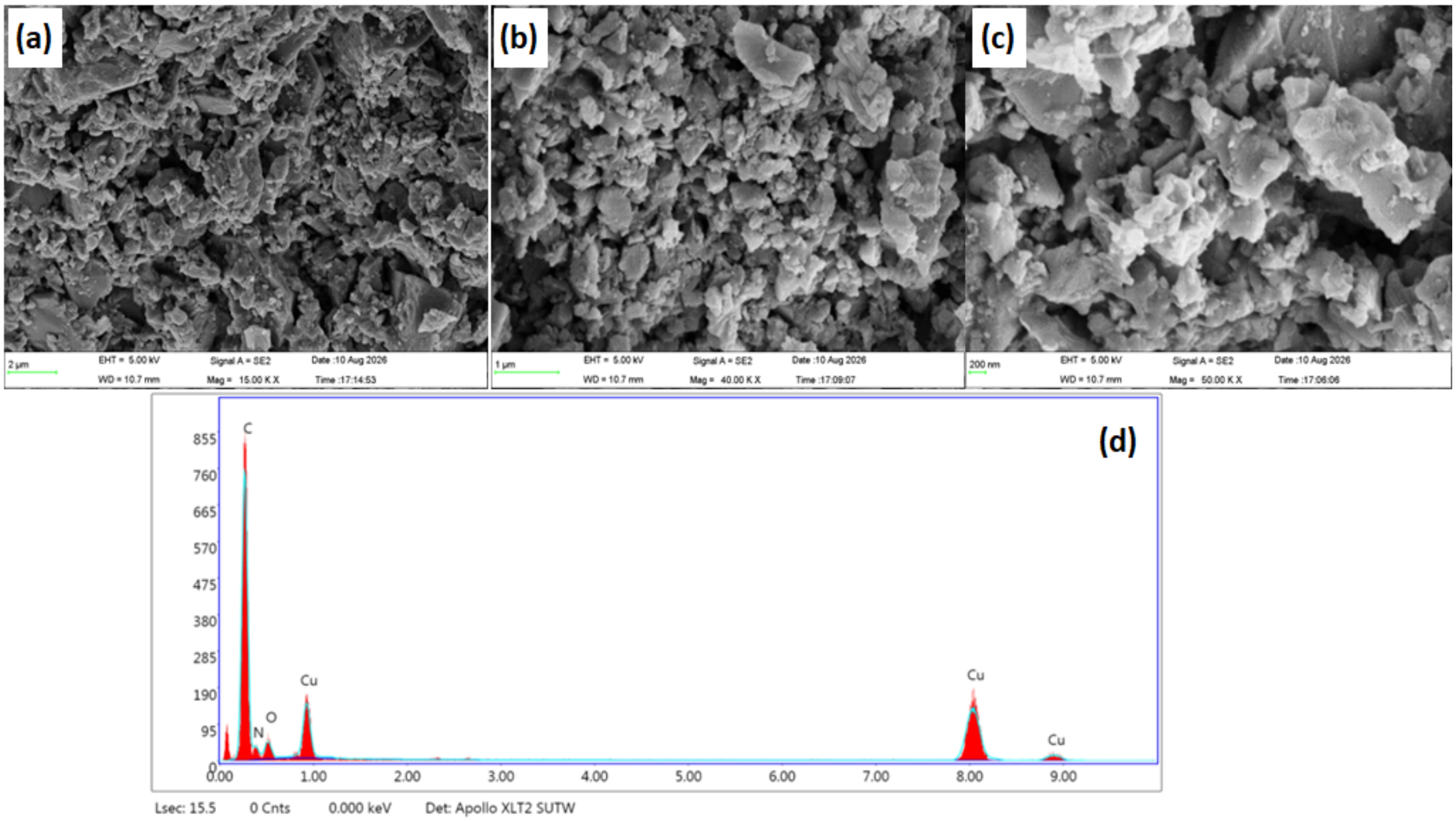

Element	Weight %	Atomic %	Net Int.	Net Error%	KABFactor
C K	59.44	83.65	761.10	1.22	1
N K	3.31	3.99	25.90	19.14	1.63
O K	3.10	3.28	33.50	18.06	1.18
CuK	34.15	9.08	315.80	2.55	1.38

Fig. S3. (a, b and c) SEM images of Rbf-Cu-MOF at different magnifications, and (d) EDX spectrum and table show the composition of various elements present in Rbf-Cu-MOF.

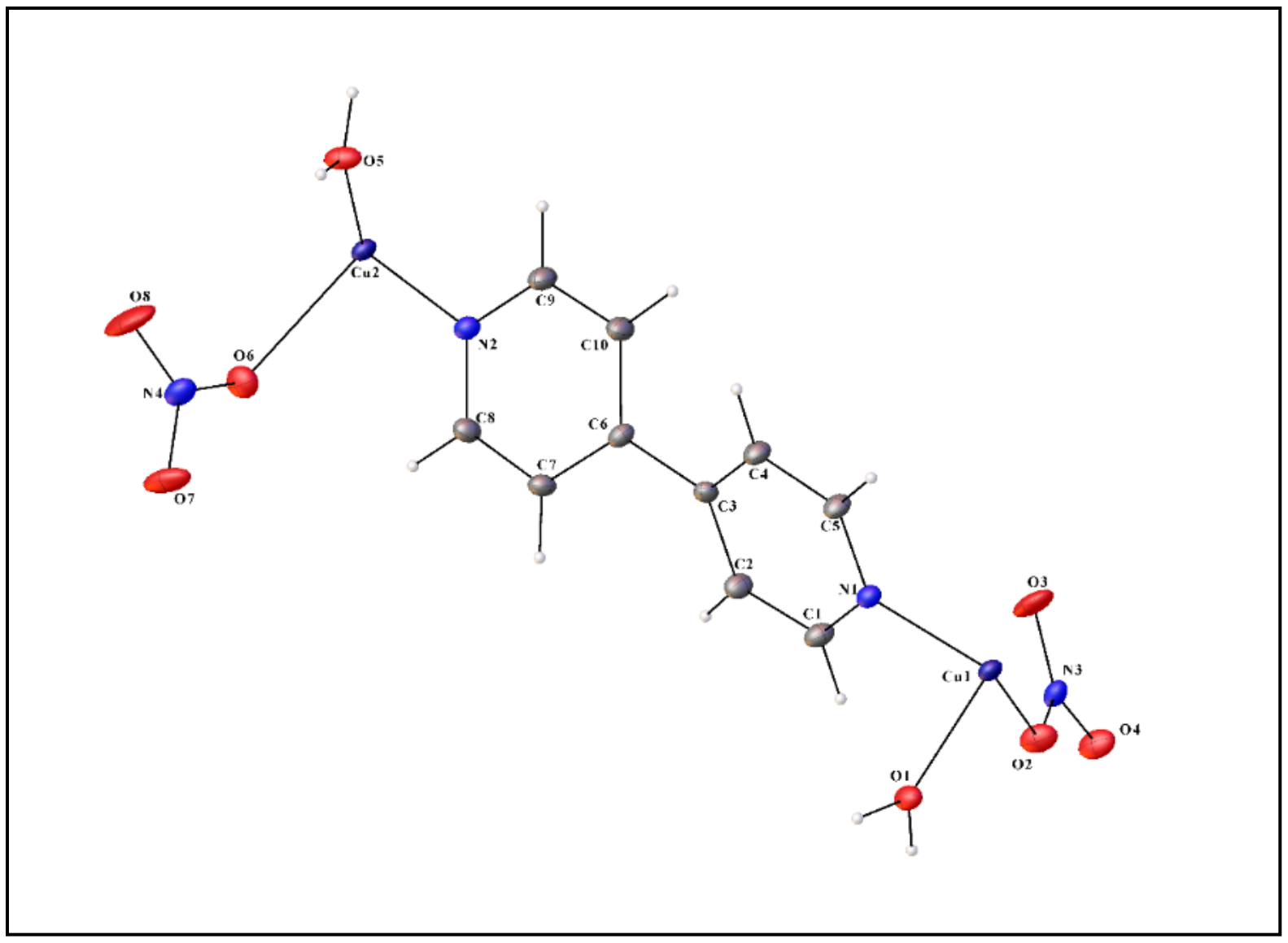

Fig. S4. Single crystal unit cell representation of pristine Cu-MOF. [1-4]

Table S1. Crystal data and structure refinement for Cu-MOF

CCDC number	2490111	
Empirical formula	$C_{10}H_{12}CuN_4O_8$	
Formula weight	379.78	
Temperature	110.0 K	
Wavelength	0.71073 Å	
Crystal system	Monoclinic	
Space group	P 1 21/c 1	
Unit cell dimensions	*a* = 18.6865(16) Å	α= 90°

	b = 11.9234(10) Å	β = 96.971(2)°
	c = 7.3841(6) Å	γ = 90°
Volume	1633.1(2) Å^3	
Z	4	
Density (calculated)	1.545 Mg/m^3	
Absorption coefficient	1.382 mm^{-1}	
F(000)	772	
Crystal size	0.341 × 0.247 × 0.134 mm^3	
Theta range for data collection	2.030 to 30.532°	
Index ranges	-26<=h<=26, -17<=k<=17, -10<=l<=10	
Reflections collected	12140	
Independent reflections	12140	
Completeness to theta = 25.242°	99.8%	
Absorption correction	Semi-empirical from equivalents	
Max. and min. transmission	0.648 and 0.518	
Refinement method	Full-matrix least-squares on F^2	

Data / restraints / parameters	12140 / 1 / 216	
Goodness-of-fit on F^2	1.057	
Final R indices [I>2sigma(I)]	R1 = 0.0329, wR2 = 0.0836	

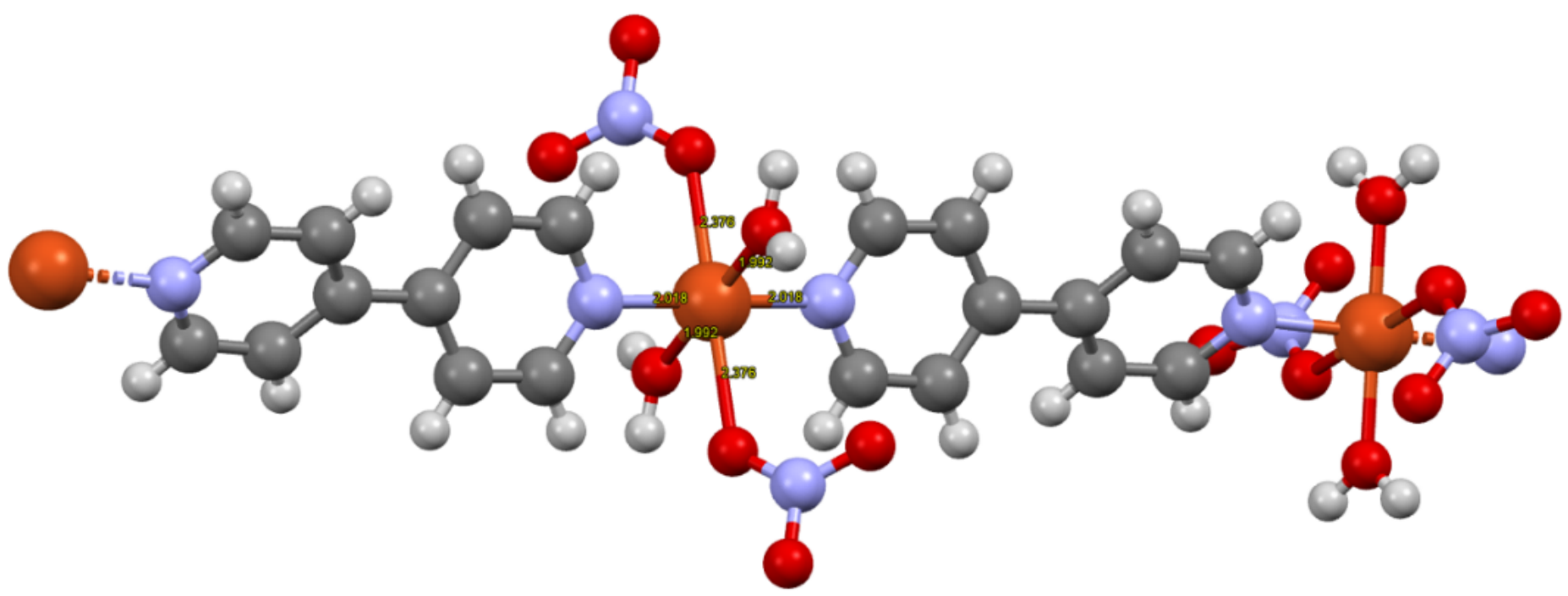

Fig. S5. Bond distances from central Cu ion.

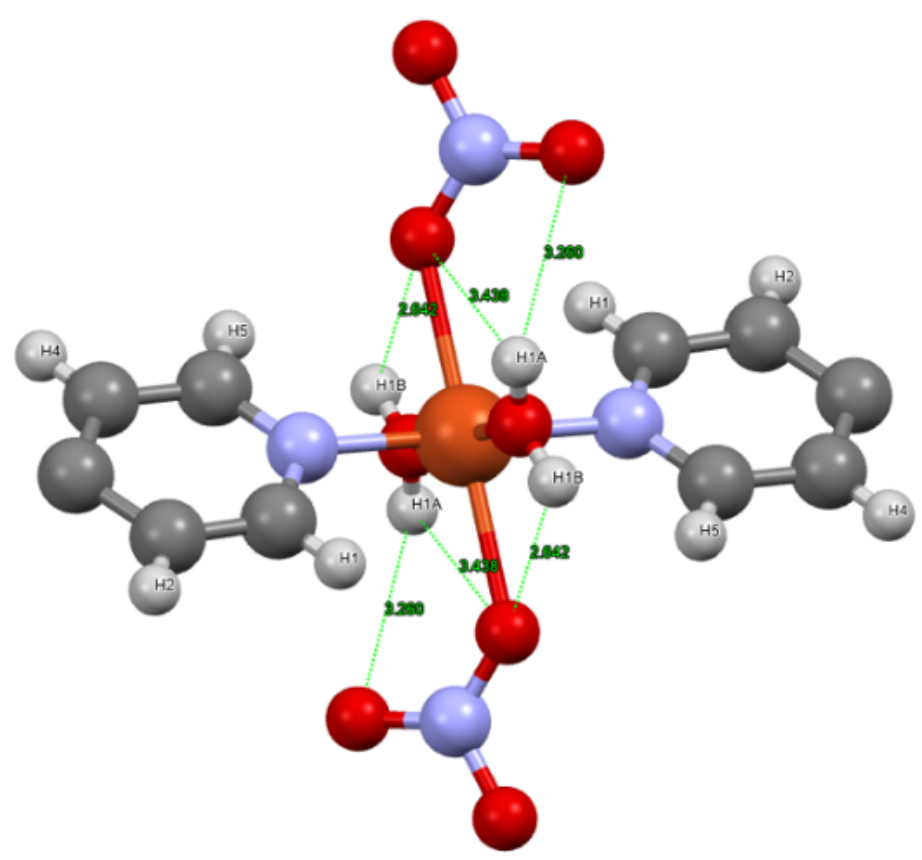

Fig. S6. Hydrogen bonding interactions between water and nitrate.

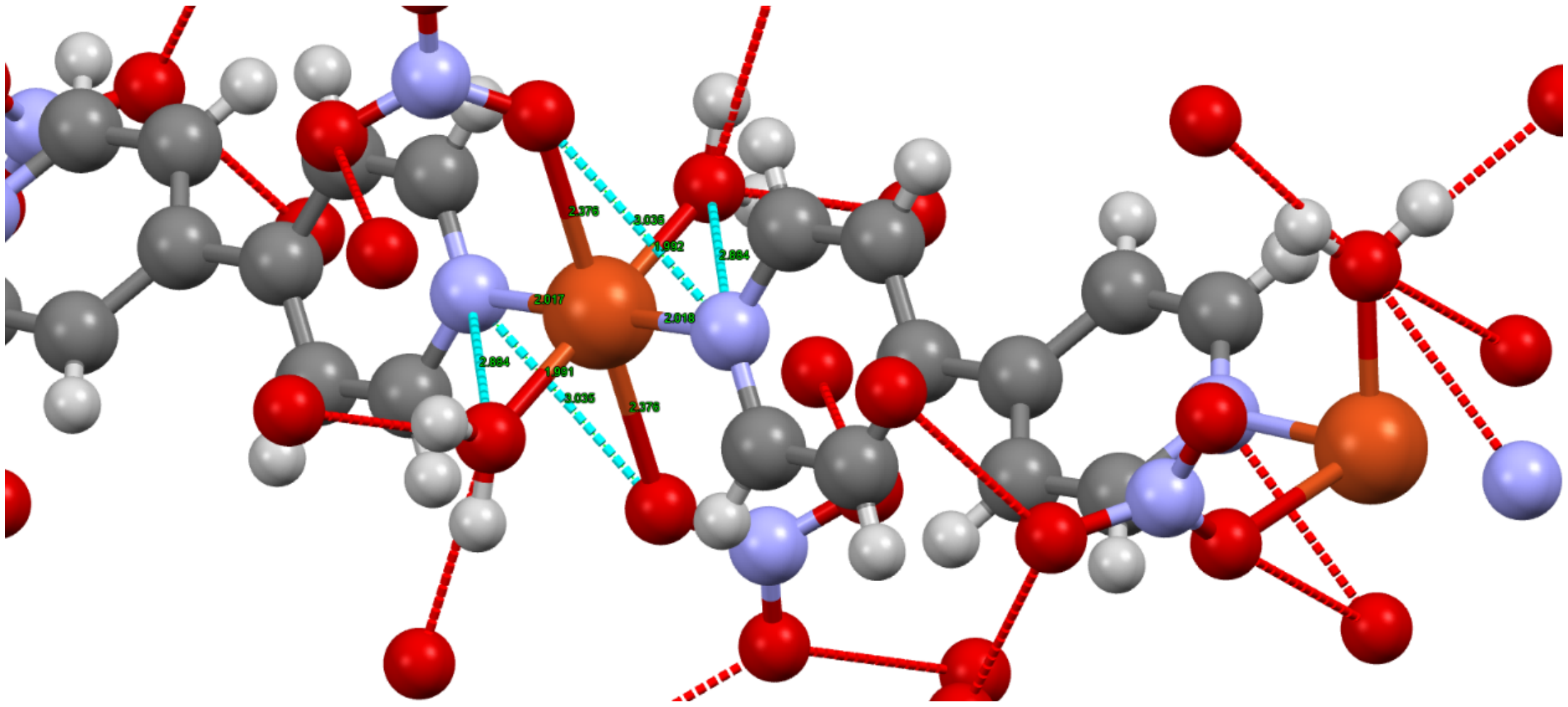

Fig. S7. Distances between nitrogen (bipyridine) and oxygen (water or nitrate).

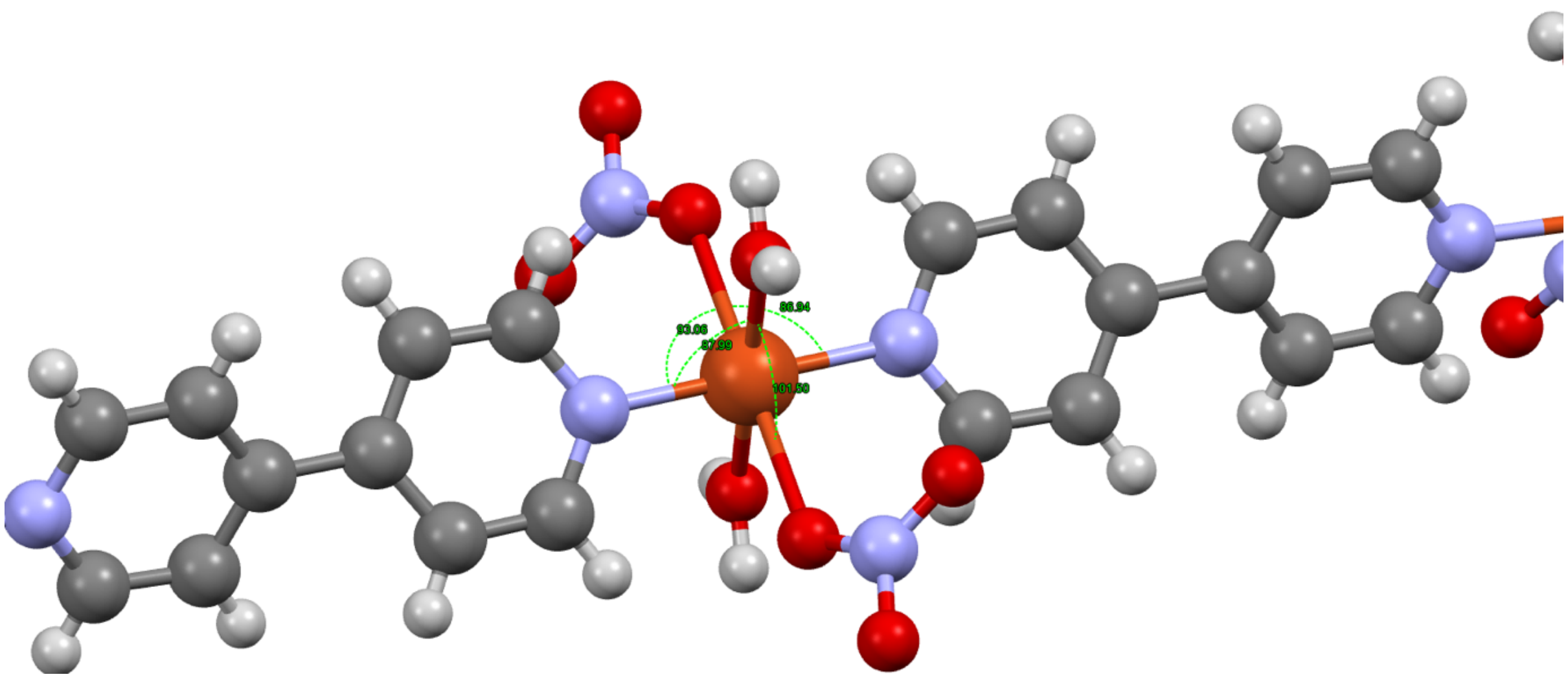

Fig. S8. Bond angles around Cu ion.

X-ray Absorption Spectroscopy

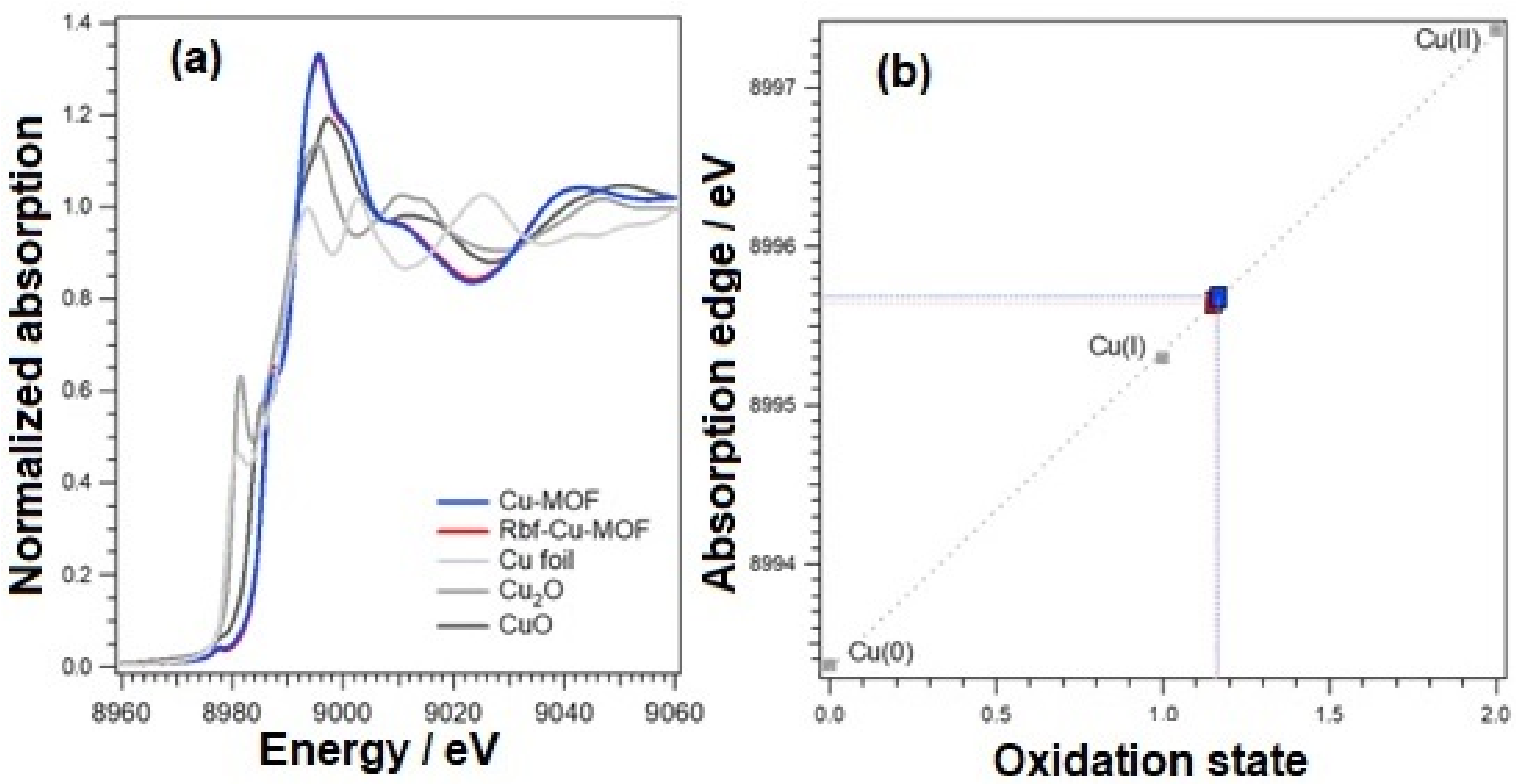

Fig. S9. (a) XANES region at the Cu K absorption edge for Cu-MOF and Rbf-Cu-MOF, and (b) Determination of oxidation state from the absorption edge. Standard samples have been included as references to determine the oxidation state.

Table S2. Fitting parameters of the *ex-situ* EXAFS data for Cu-MOF and Rbf-Cu-MOF samples using the model reported in Fig. S9

Sample	Path	N	S_o^2	R / Å	σ^2 × 10^4	R_f
Cu-MOF	Cu−O	2	0.72	1.93 ± 0.04	67 ± 15	0.025
	Cu−N	2		2.04 ± 0.05	81 ± 11	
	Cu−O	2		2.63 ± 0.10	211 ± 112	
	Cu−C	4		2.91 ± 0.04	46 ± 29	
Rbf-Cu-MOF	Cu−O	2	0.76	1.94 ± 0.05	83 ± 31	0.022
	Cu−N	2		2.04 ± 0.05	117 ± 33	

	Cu−O	2		2.62 ± 0.11	428 ± 276	
	Cu−C	4		2.92 ± 0.03	41 ± 20	

FT-IR Spectroscopy

FT-IR measurements were carried out by using a Thermo Scientific Nicolet iS5 FT-IR instrument. Fig. S10 shows the FT-IR spectra of both the samples, as well as those of 4,4'-bpy and Rbf. In the spectrum of Cu-MOF, peaks were observed at 3442, 1610, 1417 and 1383 cm$^{−1}$, which corresponded to the O−H, conjugated C=C, C−N and N−O stretching frequencies, respectively.[5,6] In the spectrum of Rbf-Cu-MOF, in addition to the peaks observed for Cu-MOF, two new peaks at 1724 and 1578 cm$^{−1}$ were present. These peaks could be related to the carbonyl stretching and N−H asymmetric bending vibrations, respectively, of Rbf.

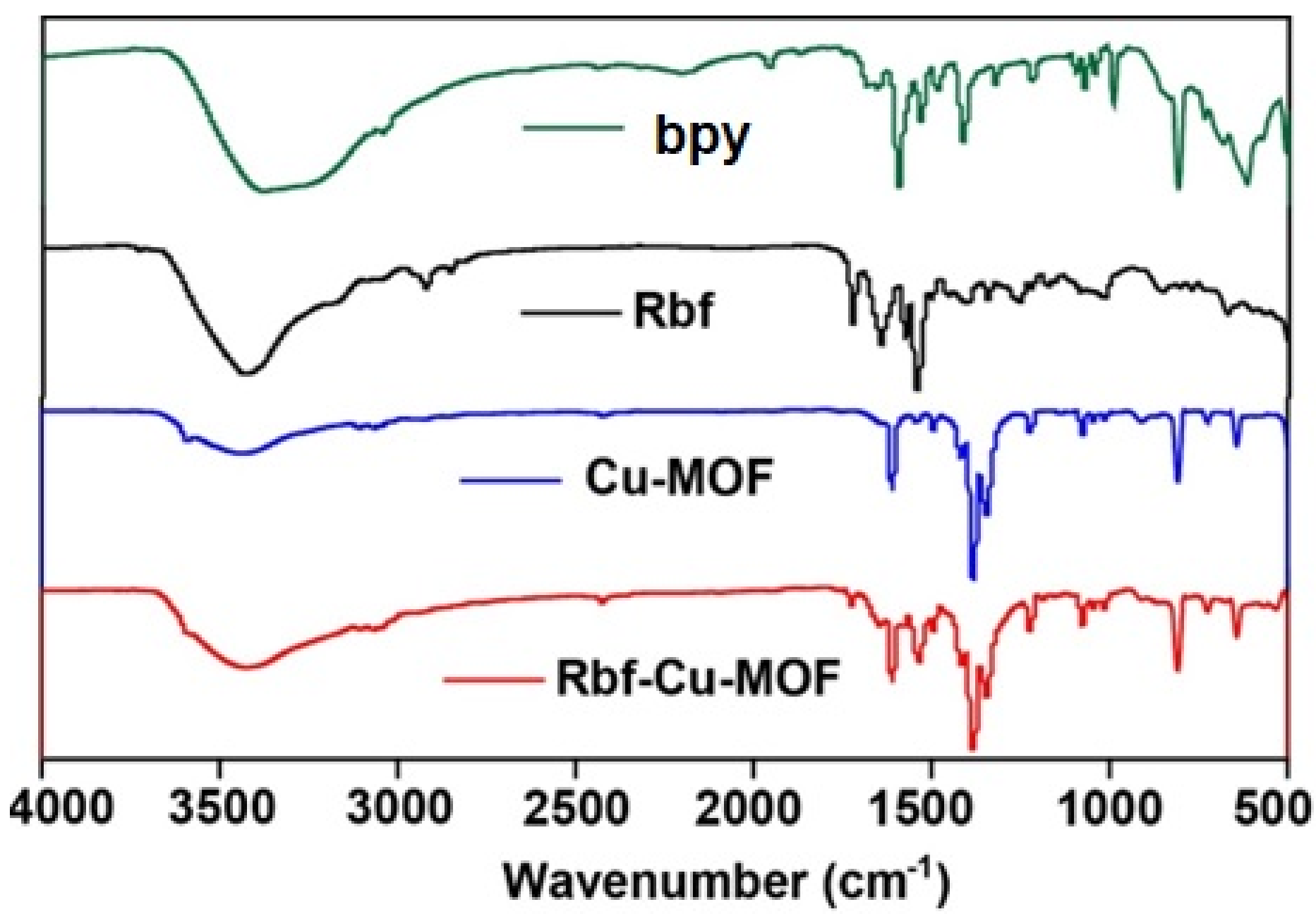

Fig. S10. FT-IR spectra of Cu-MOF, Rbf-Cu-MOF, Rbf and 4,4'-bpy.

UV-Vis Spectroscopy

UV-Vis spectroscopy was performed by using a Shimadzu-2600 spectrophotometer. UV-Vis spectra of Cu-MOF and Rbf-Cu-MOF were recorded in water (Fig. S11). In the spectrum of Cu-MOF, strong absorption bands at 224, 234 and 268 nm were observed, corresponding to π–π* transitions of the bpy ligand. In the case of Rbf-Cu-MOF, in addition to the bands observed for Cu-MOF, additional absorption bands at 373 and 446 nm, corresponding to n–π* transitions, were observed. These bands were related to the presence of Rbf, as deduced from the comparison with the spectrum of Rbf, indicating the successful incorporation of Rbf onto the Cu-MOF structure.

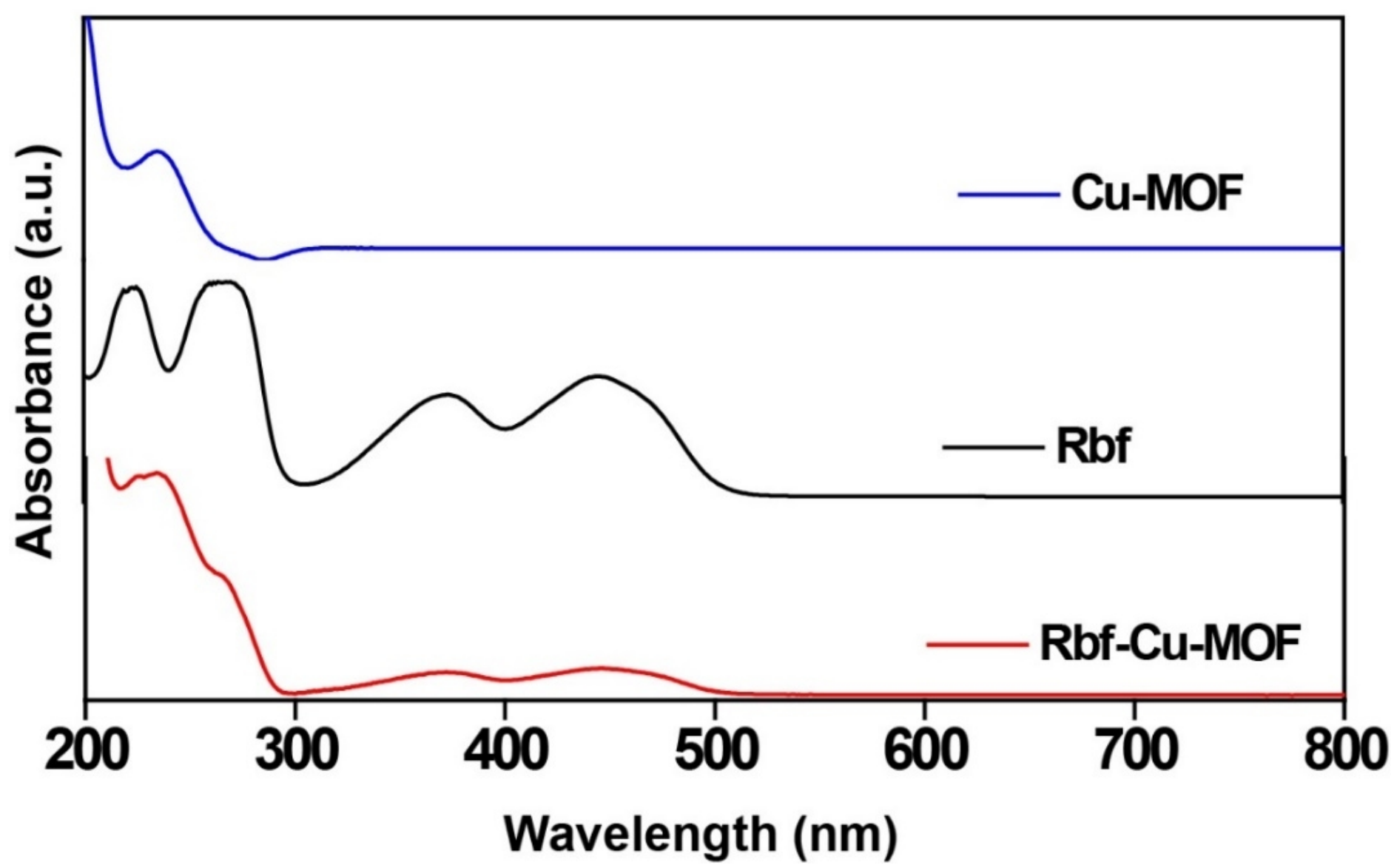

Fig. S11. UV-Vis spectra of Cu-MOF, Rbf and Rbf-Cu-MOF.

N_2 Physisorption and Surface Area Analysis

Nitrogen adsorption-desorption experiments were carried out at 77 K using a Quantachrome Quadrasorb automatic volumetric instrument. The samples were degassed at room temperature for 24 h, and at 100 °C for 36 h under ultrahigh vacuum (10^{-8} mbar) for gas adsorption measurements. Fig. S12 reports the isotherms for Cu-MOF and Rbf-Cu-MOF. Both the isotherms are type I, suggesting a microporous nature. Cu-MOF displayed a BET surface area of 76 m^2/g, while Rbf-Cu-MOF presented a BET surface area of 40 m^2/g. The decrease in the surface area could be attributed to the presence of Rbf, blocking the porosity of the MOF structure.[6]

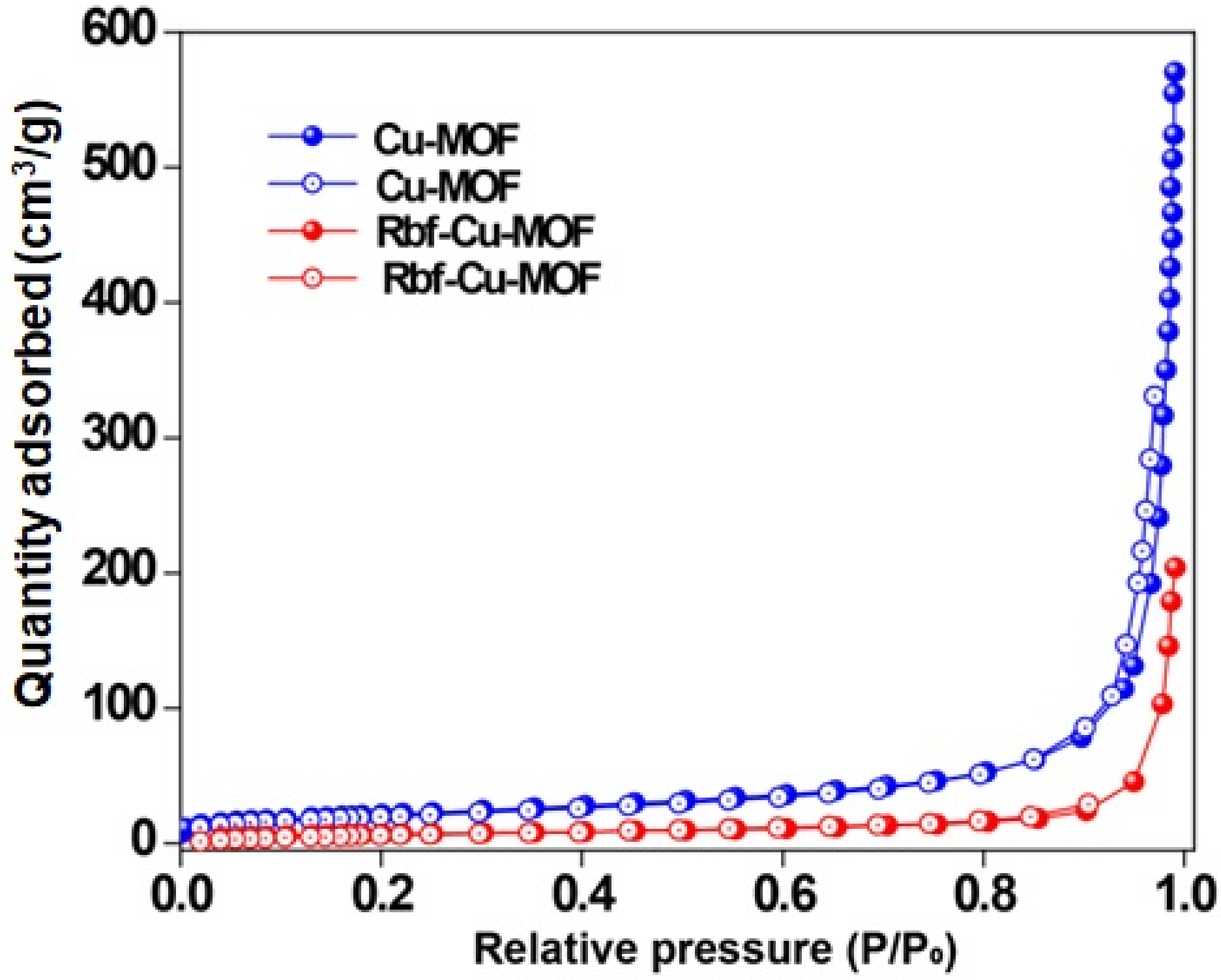

Fig. S12. N_2 adsorption-desorption isotherms of Cu-MOF and Rbf-Cu-MOF.

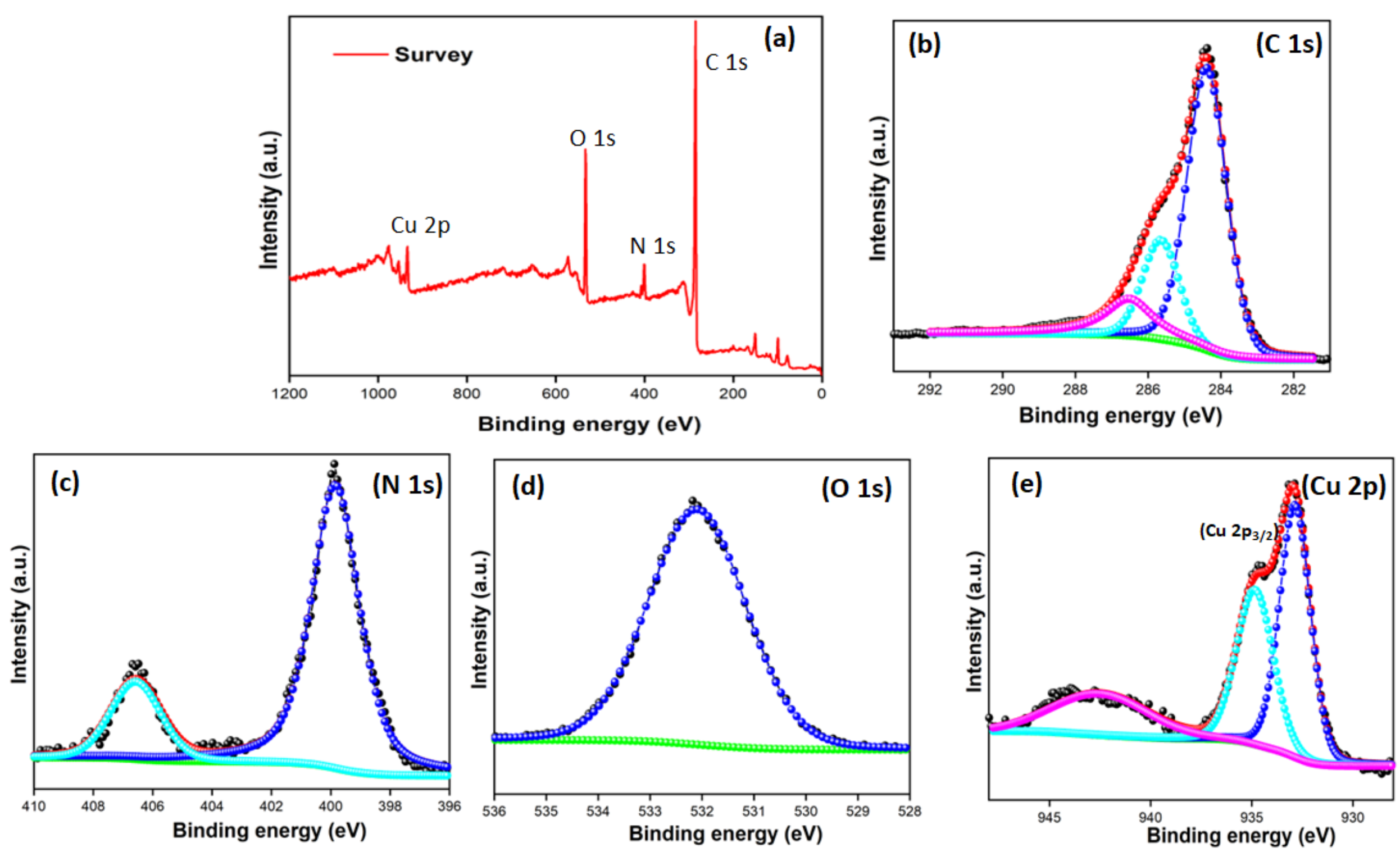

Fig. S13. XPS spectra of Cu-MOF. (a) Survey, (b) C 1s, (c) N 1s, (d) O 1s and (e) Cu2p spectra.

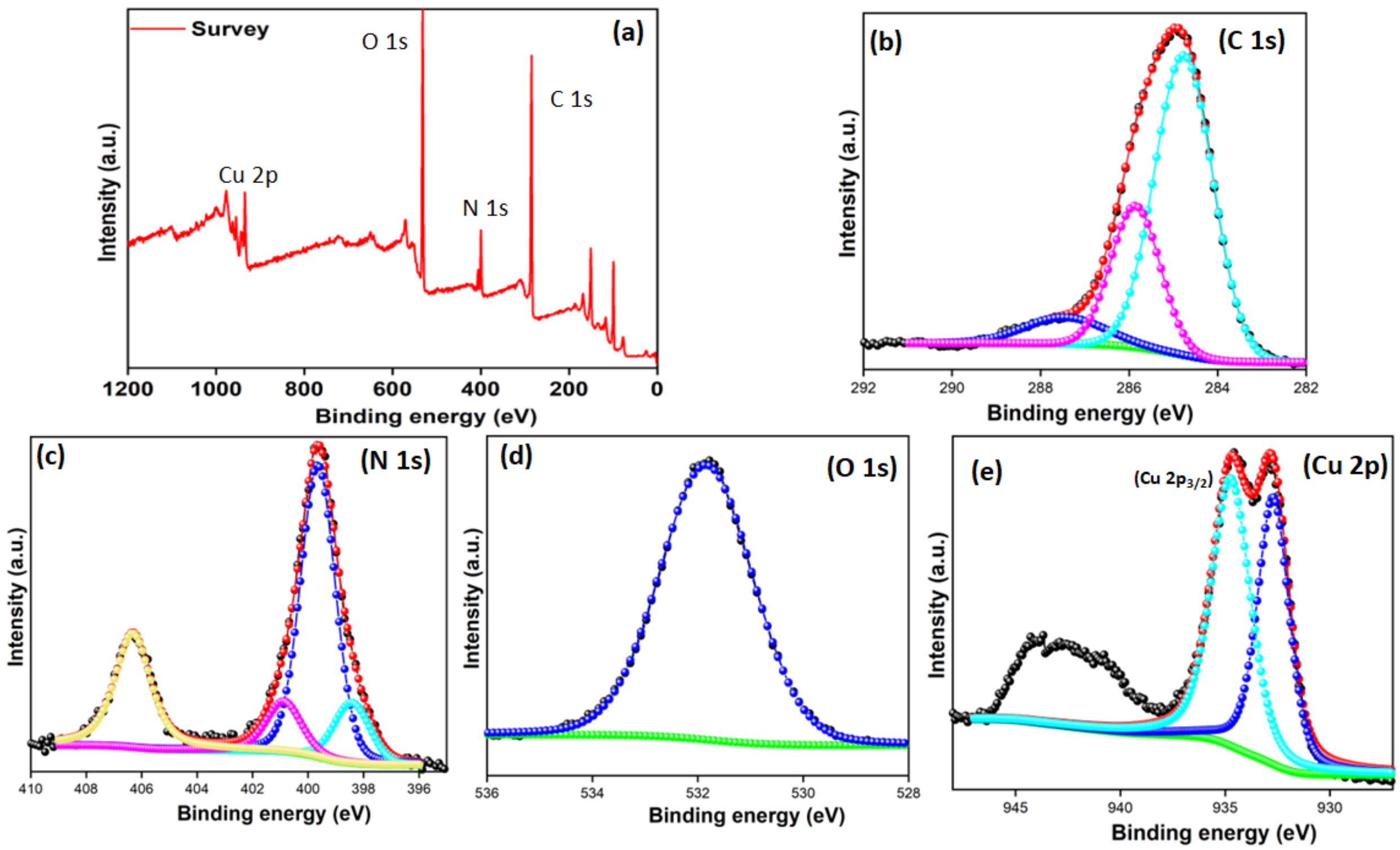

Fig. S14. XPS spectra of Rbf-Cu-MOF. (a) Survey, (b) C 1s, (c) N 1s, (d) O 1s and (e) Cu2p spectra.

Electrochemical Studies

All the electrochemical analyses were performed using an Autolab PGSTAT 30 (Ecochemie) instrument equipped with a standard three-electrode system. Ag/AgCl (in saturated KCl) and graphite rod were used as reference and counter electrodes, respectively. The working electrode used was a glassy carbon (GC, 3 mm diameter) embedded in Teflon. This electrode was polished on a polishing cloth using 0.3 μm alumina slurry followed by washing with water and acetone. The sample coating on the working electrode was carried out as follows: 5 mg of the catalyst was dispersed in 1 mL of ethanol-water (3:2) solution by sonication. 5 μL aliquot of the prepared slurry was drop casted on the GC electrode

with the help of a micro syringe.[6] Subsequently, 3 μL of 0.1% Nafion® solution in ethanol was coated on the surface as a binder.

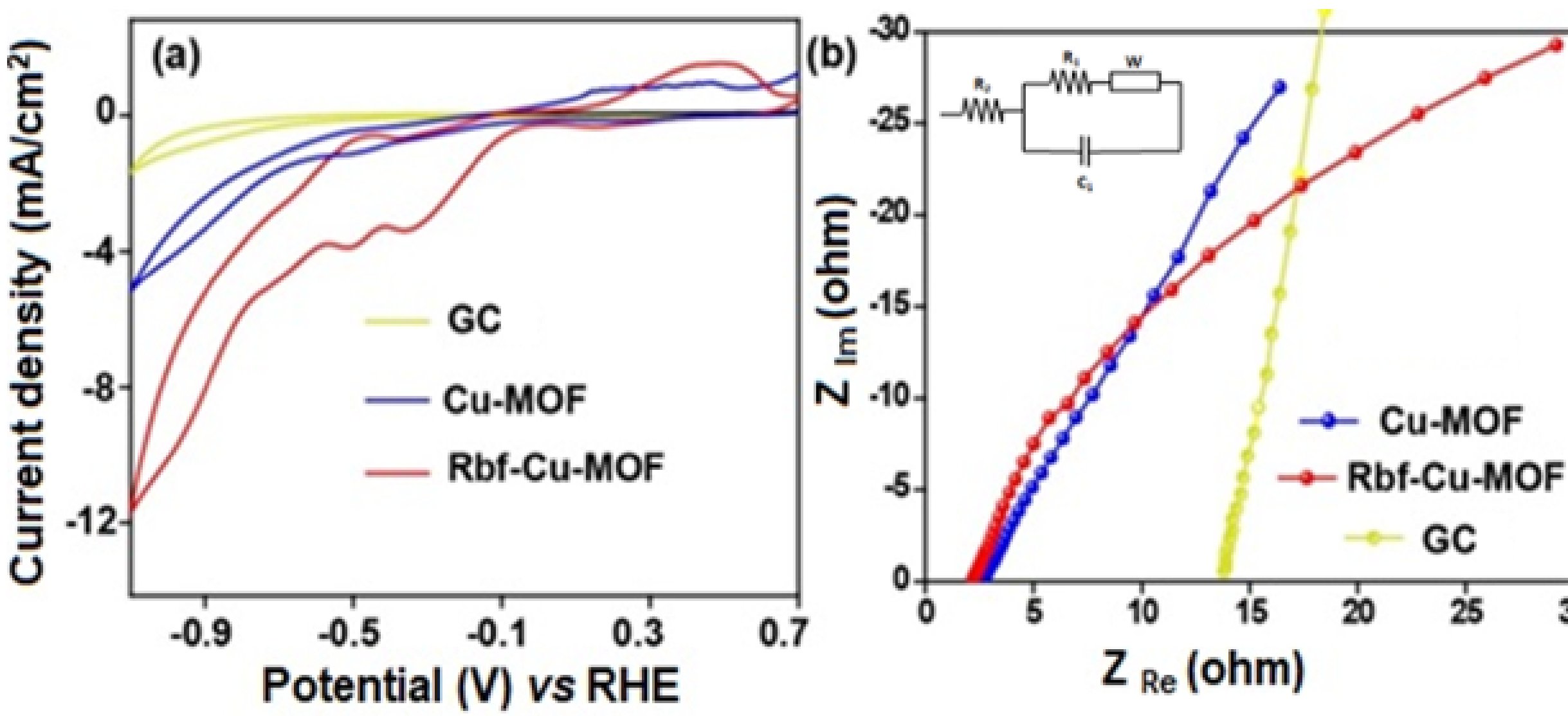

Fig. S15. (a) CVs performed in CO_2 saturated 0.5 M $NaHCO_3$ at a sweep rate of 50 mV/s, and (b) impedance analyses of GC, Cu-MOF and Rbf-Cu-MOF.

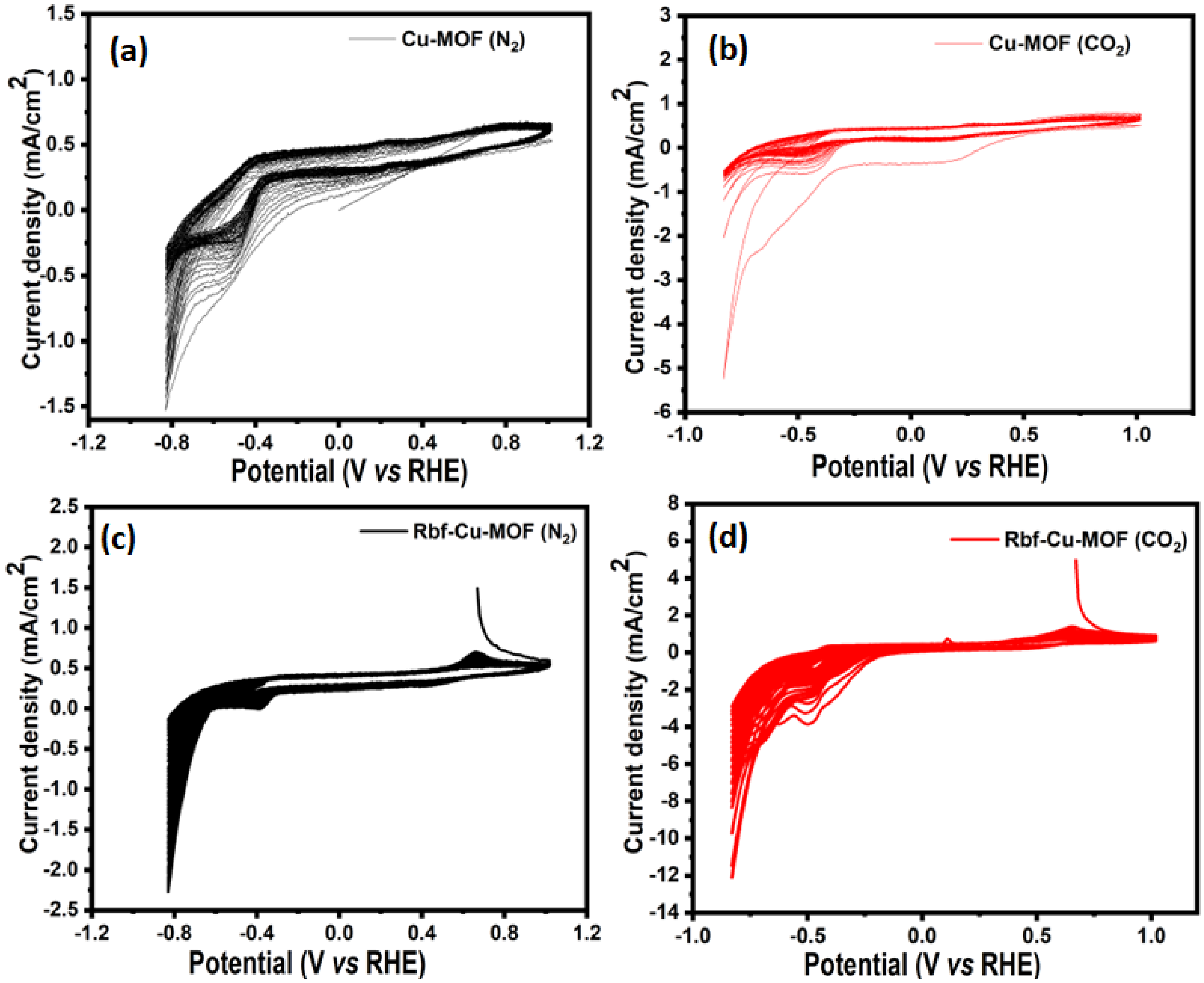

Fig. S16. Multiple-cycle CVs of Cu-MOF and Rbf-Cu-MOF recorded in (a, c) N₂- and (b, d) CO₂-saturated 0.5 M $NaHCO_3$ at a scan rate of 50 mV/s.

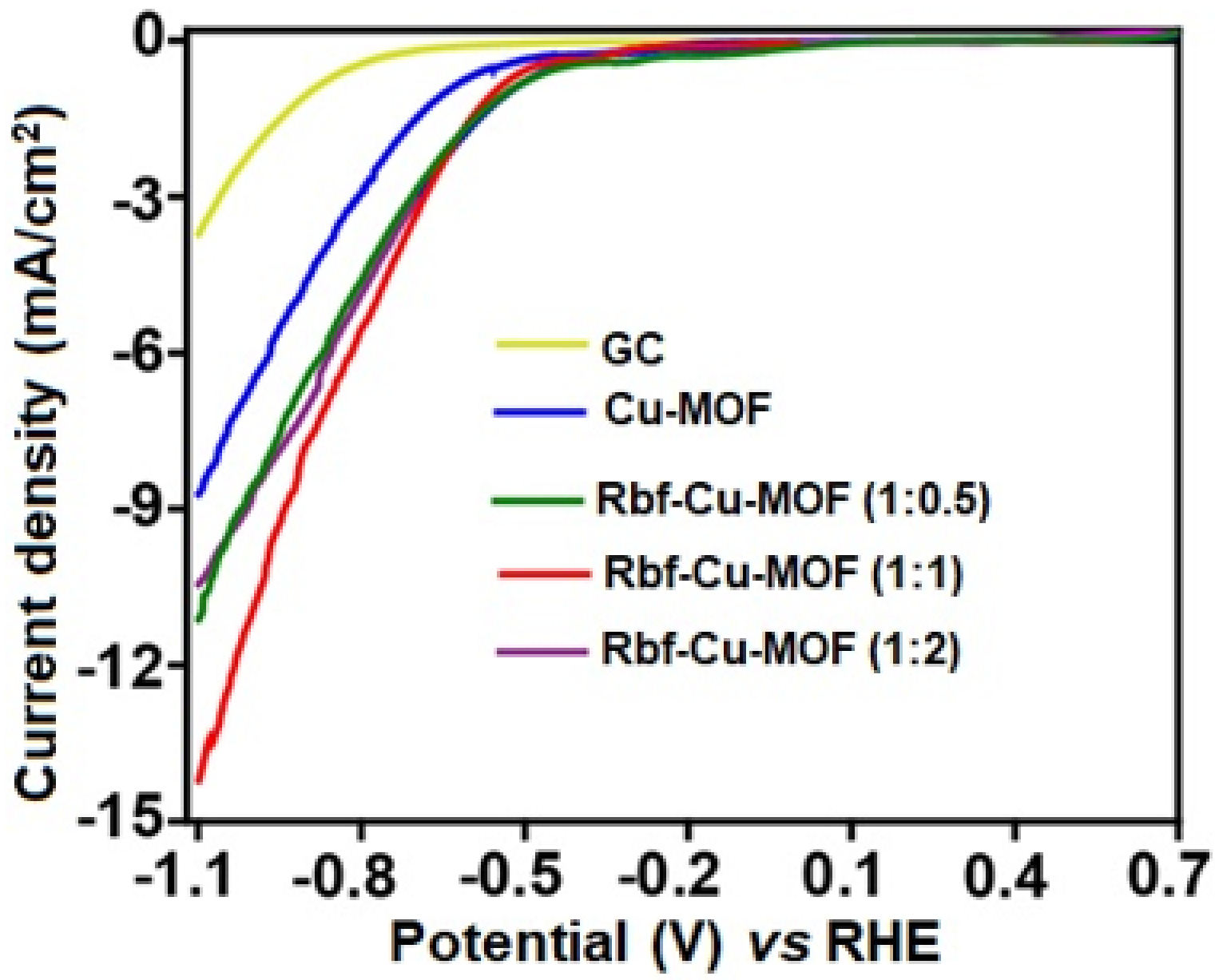

Fig. S17. LSVs performed in CO_2-saturated 0.5 M $NaHCO_3$ at a sweep rate of 10 mV/s on GC, Cu-MOF, Rbf-Cu-MOF (1:0.5), Rbf-Cu-MOF (1:1), and Rbf-Cu-MOF (1:2) (The values in the parenthesis represent feed molar ratios of Cu-MOF and Rbf).

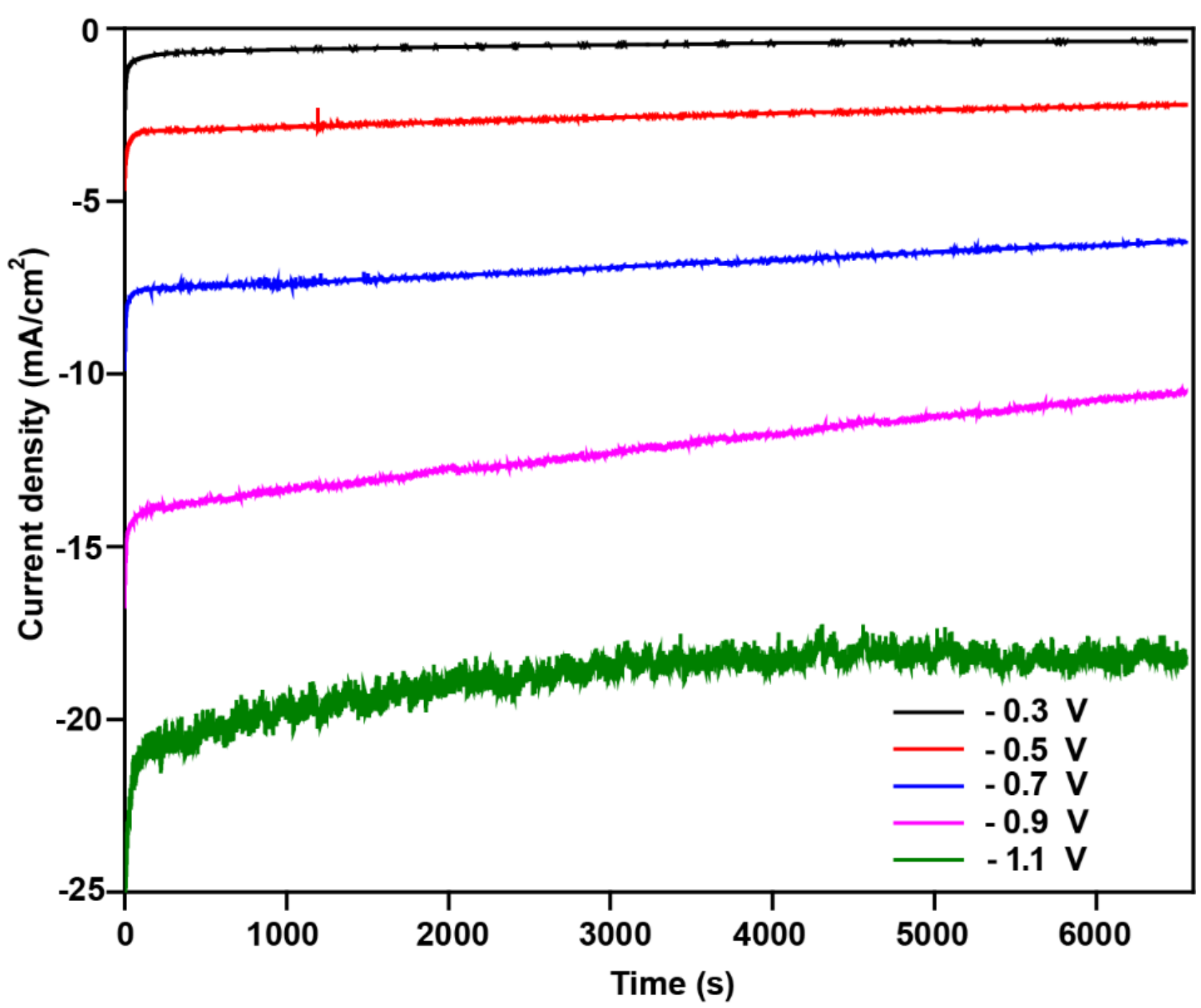

Fig. S18. Chronoamperometric responses of Rbf-Cu-MOF (1:1) at different potentials (−0.3

to −1.1 V *vs* RHE) for 2 h.

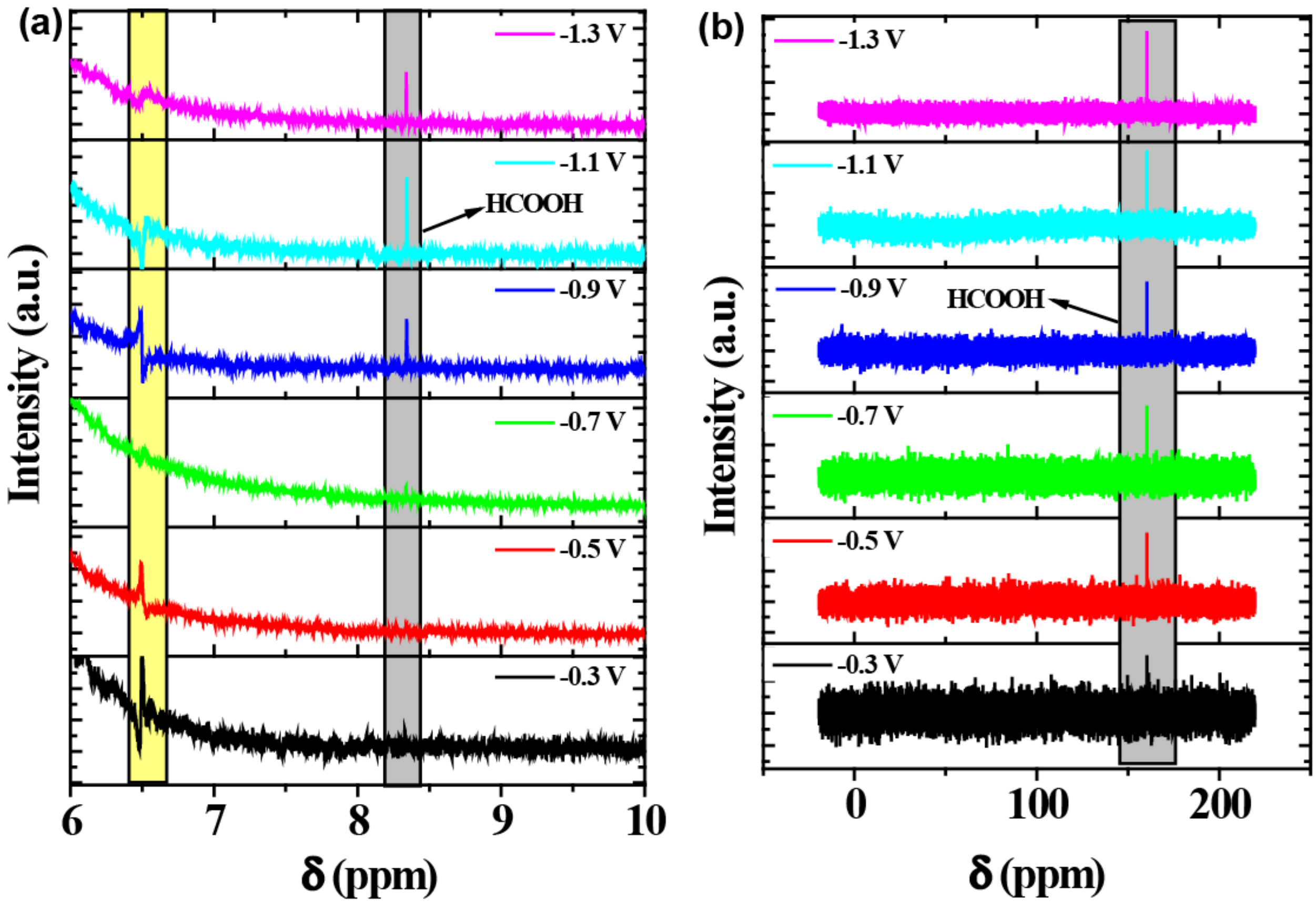

Fig. S19. (a, b) ^{1}H- and ^{13}C-NMR spectra of formic acid, which were recorded after electrolysis on Cu-MOF modified GC at various potentials (−0.3 to −1.3 V *vs* RHE) for 2 h in CO_2 saturated 0.1 M $NaHCO_3$.

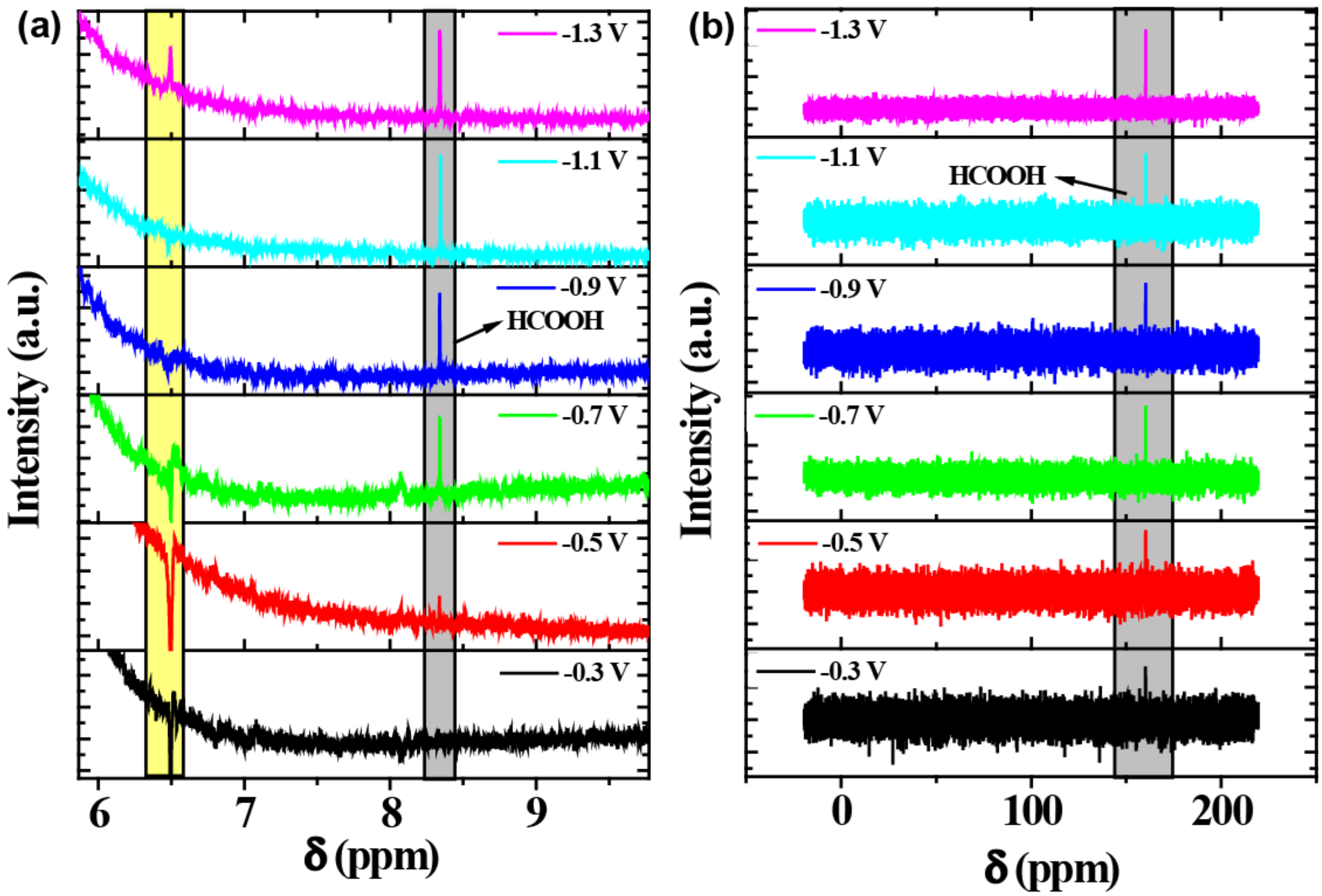

Fig. S20. (a, b) ^{1}H- and ^{13}C-NMR spectra of formic acid, which were recorded after electrolysis on Rbf-Cu-MOF (1:1) modified GC at various potentials (−0.3 to −1.3 V *vs* RHE) for 2 h in CO_2 saturated 0.1 M $NaHCO_3$.

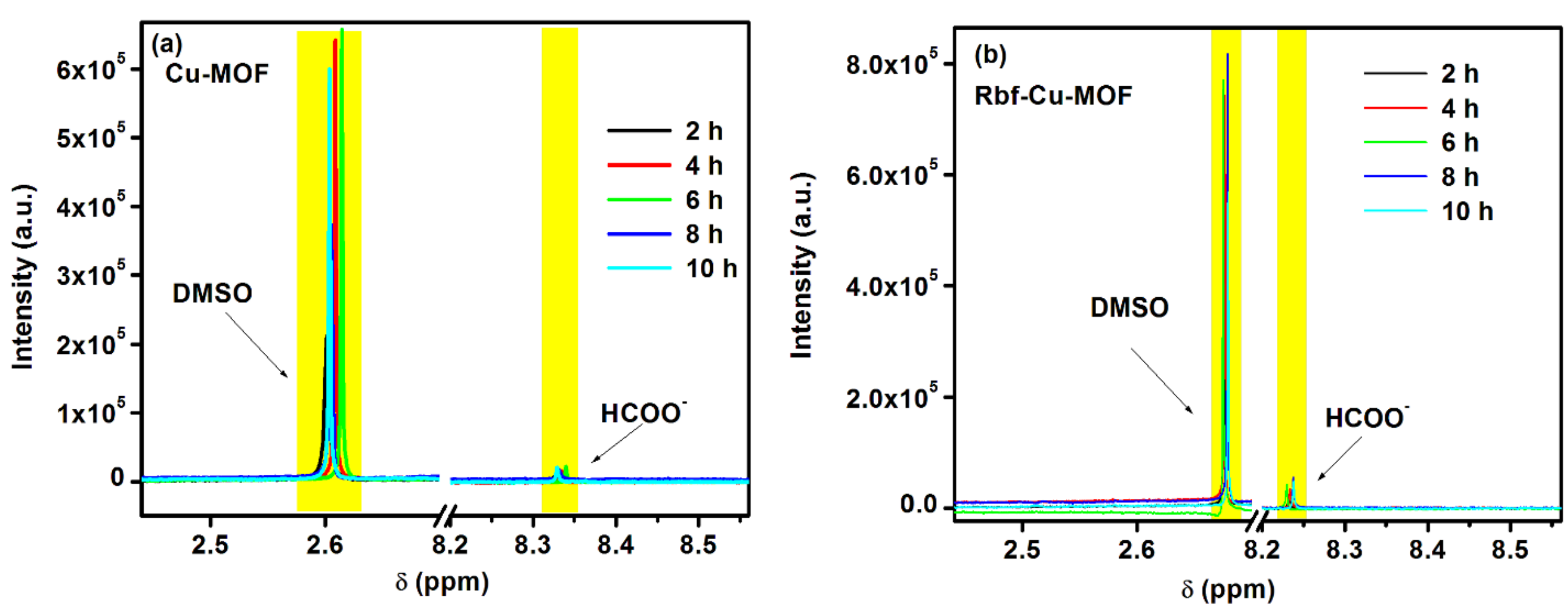

Fig. S21. (a, b) ^{1}H-NMR spectra for CO_2 reduction using Cu-MOF and Rbf-Cu-MOF (1:1) catalysts in CO_2 saturated 0.1 M $NaHCO_3$ at −1.1 V (*vs* RHE) for various times of electrolysis.

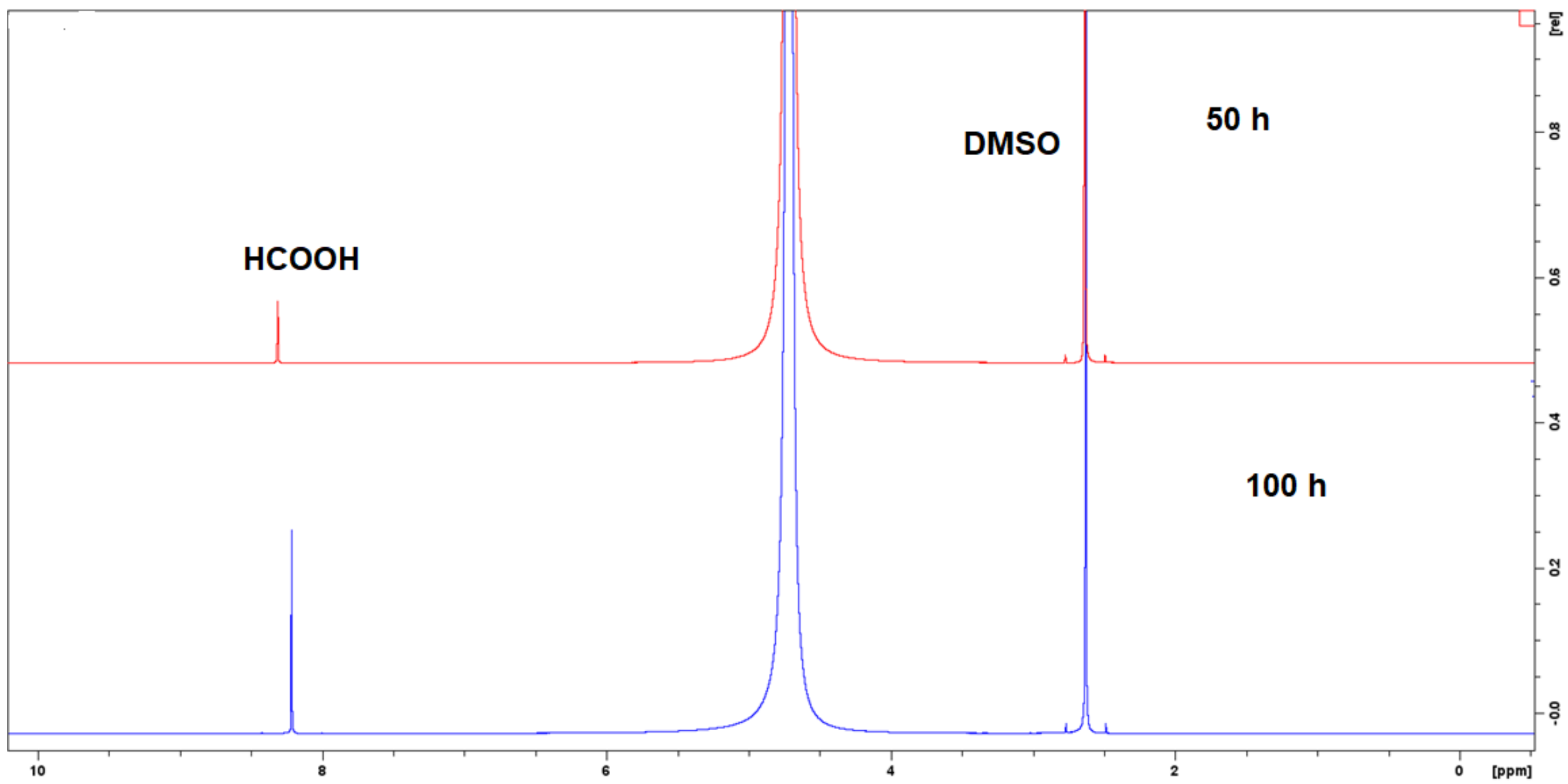

Fig. S22. ^{1}H-NMR spectra for CO_2 reduction using Rbf-Cu-MOF (1:1) catalyst in CO_2 saturated 0.1 M $NaHCO_3$ at −1.1 V (*vs* RHE) for 50 and 100 h of electrolysis.

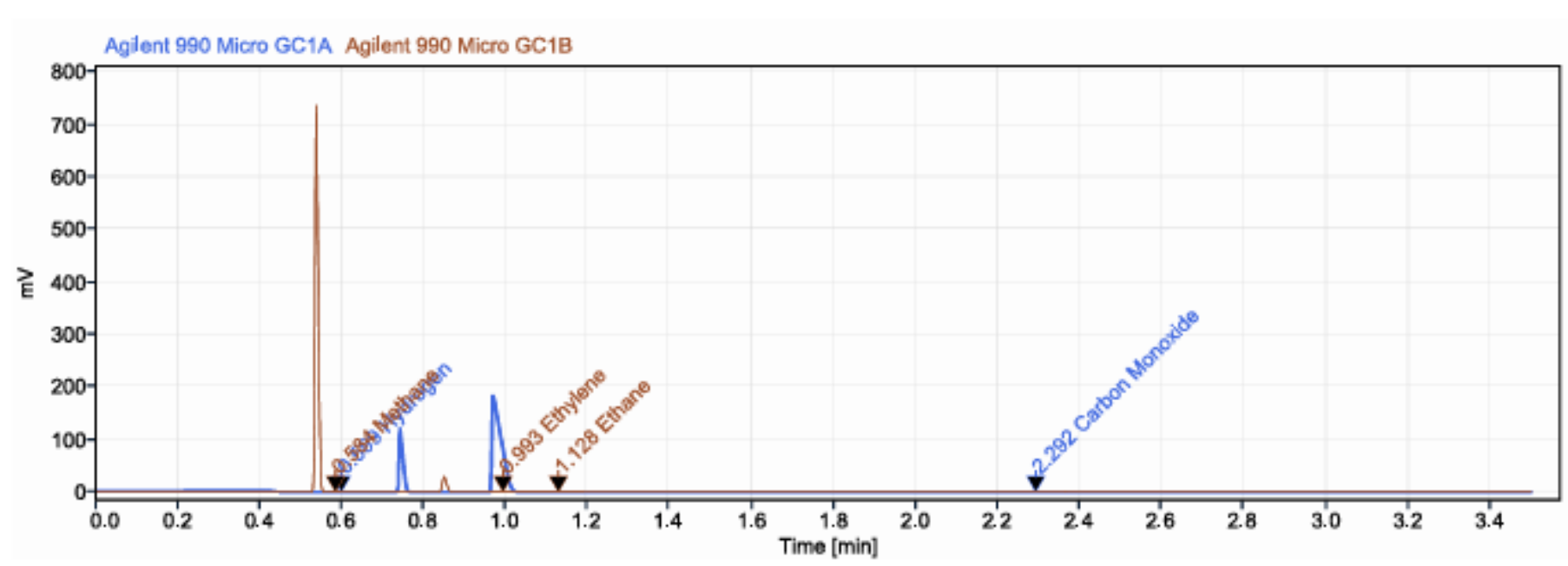

Signal: Agilent 990 Micro GC1A

Name	RT [min]	RF	Area	Amount [PPM]	Concentration [PPM]	Group
Hydrogen	0.60	0.000	0.004	221.198	221.198	
Carbon Monoxide	2.29	0.001	0.098	107.681	107.681	
			**Sum**	**328.879**		

Signal: Agilent 990 Micro GC1B

Name	RT [min]	RF	Area	Amount [PPM]	Concentration [PPM]	Group
Methane	0.58	0.001	0.012	14.191	14.191	
Ethylene	0.99	0.001	0.031	26.281	26.281	
Ethane	1.13	0.001	0.033	26.384	26.384	
			**Sum**	**66.857**		

Fig. S23. Gas chromatrography analysis for CO_2 reduction using Cu-MOF (1:1) catalyst in CO_2 saturated 0.1 M $NaHCO_3$ at −1.1 V (*vs* RHE).

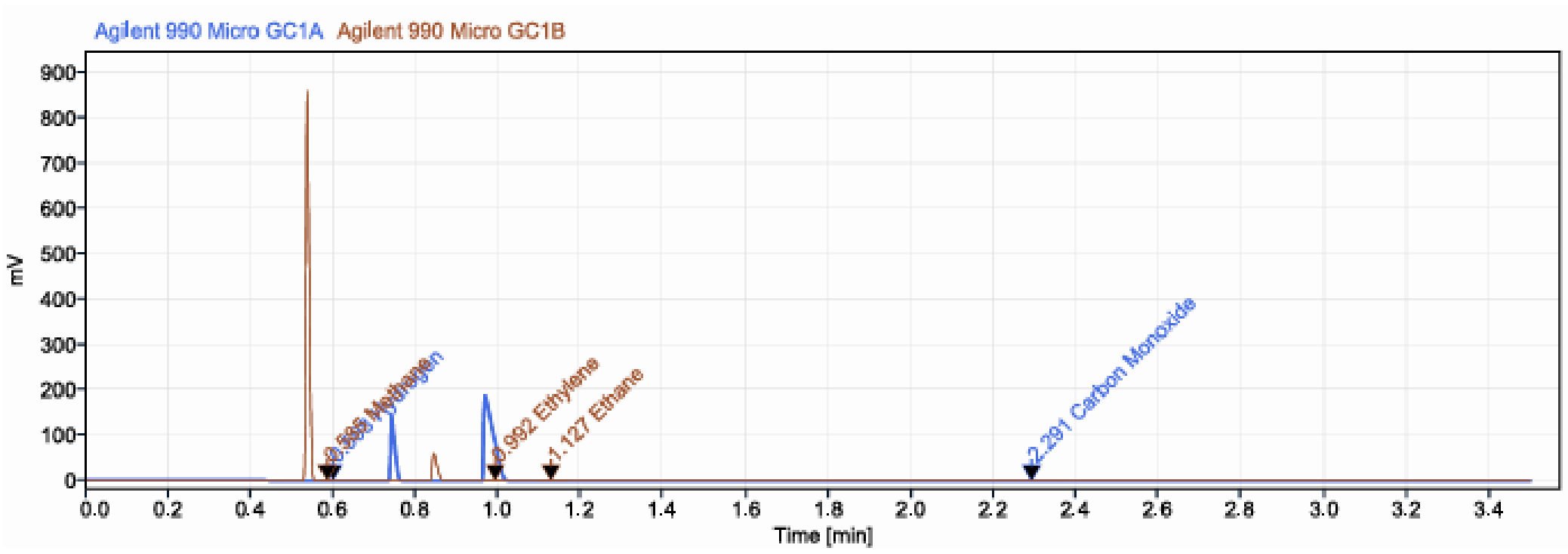

Signal: Agilent 990 Micro GC1A

Name	RT [min]	RF	Area	Amount [PPM]	Concentration [PPM]	Group
Hydrogen	0.60	0.000	0.004	203.032	203.032	
Carbon Monoxide	2.29	0.001	0.246	268.740	268.740	
			**Sum**	**471.772**		

Signal: Agilent 990 Micro GC1B

Name	RT [min]	RF	Area	Amount [PPM]	Concentration [PPM]	Group
Methane	0.58	0.001	0.005	6.215	6.215	
Ethylene	0.99	0.001	0.017	14.596	14.596	
Ethane	1.13	0.001	0.009	7.361	7.361	
			**Sum**	**28.172**		

Fig. S24. Gas chromatrography analysis for CO_2 reduction using Rbf-Cu-MOF (1:1) catalyst in CO_2 saturated 0.1 M $NaHCO_3$ at −1.1 V (*vs* RHE).

Stability under CO_2RR conditions

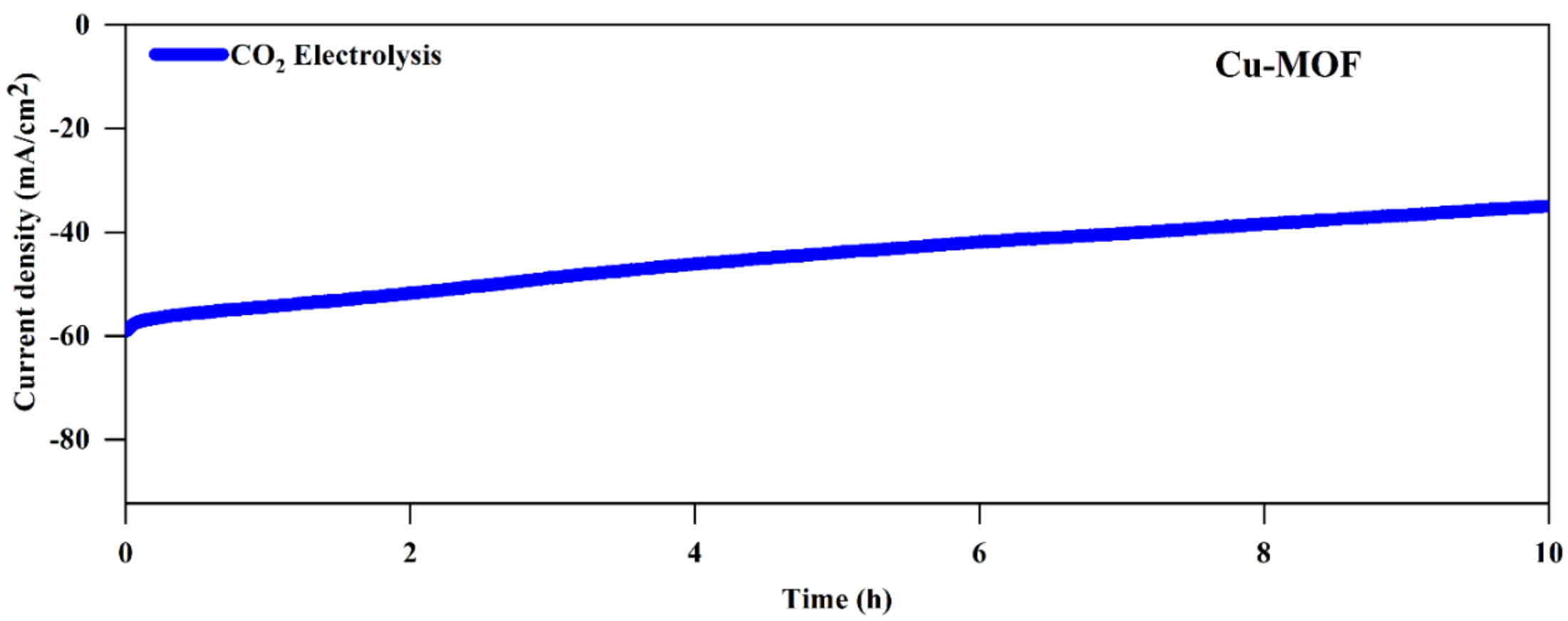

Fig. S25. Chronoamperometric stability of Cu-MOF at −1.1 (V *vs* RHE) for 10 h of continuous electrolysis.

Table S3. Fitting parameters of the *ex-situ* EXAFS data for Rbf-Cu-MOF sample after electrolysis

Sample	Path	N	S_o^2	R / Å	$\sigma^2 \times 10^4$	R_f
Rbf-Cu-MOF	Cu–O	2	0.83	1.93 ± 0.05	78 ± 38	0.016
	Cu–N	2		2.02 ± 0.05	119 ± 41	
	Cu–O	2		2.38 ± 0.08	149 ± 116	
	Cu–C	4		2.91 ± 0.04	61 ± 45	

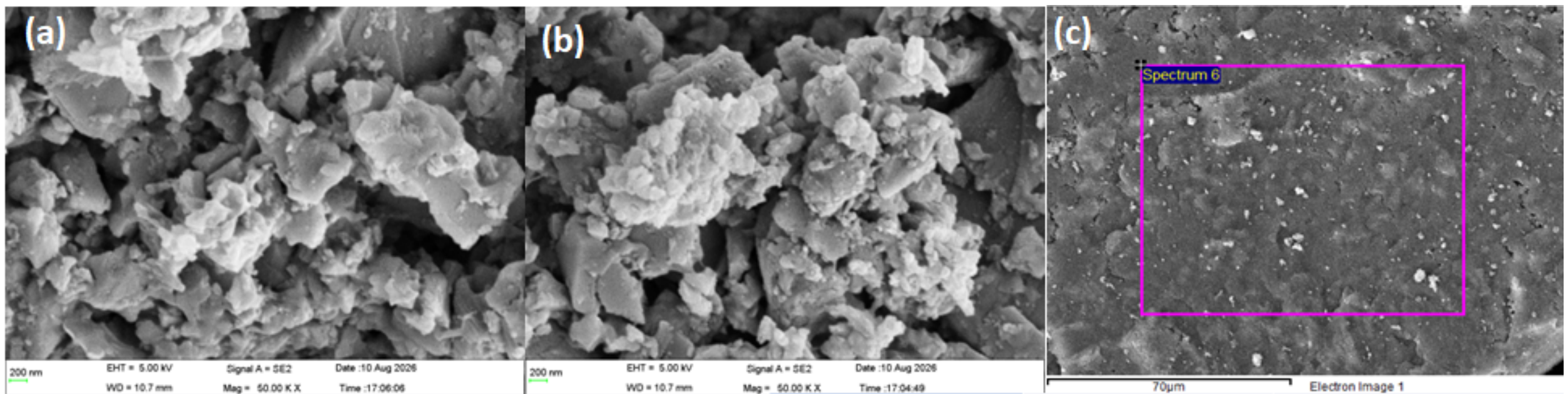

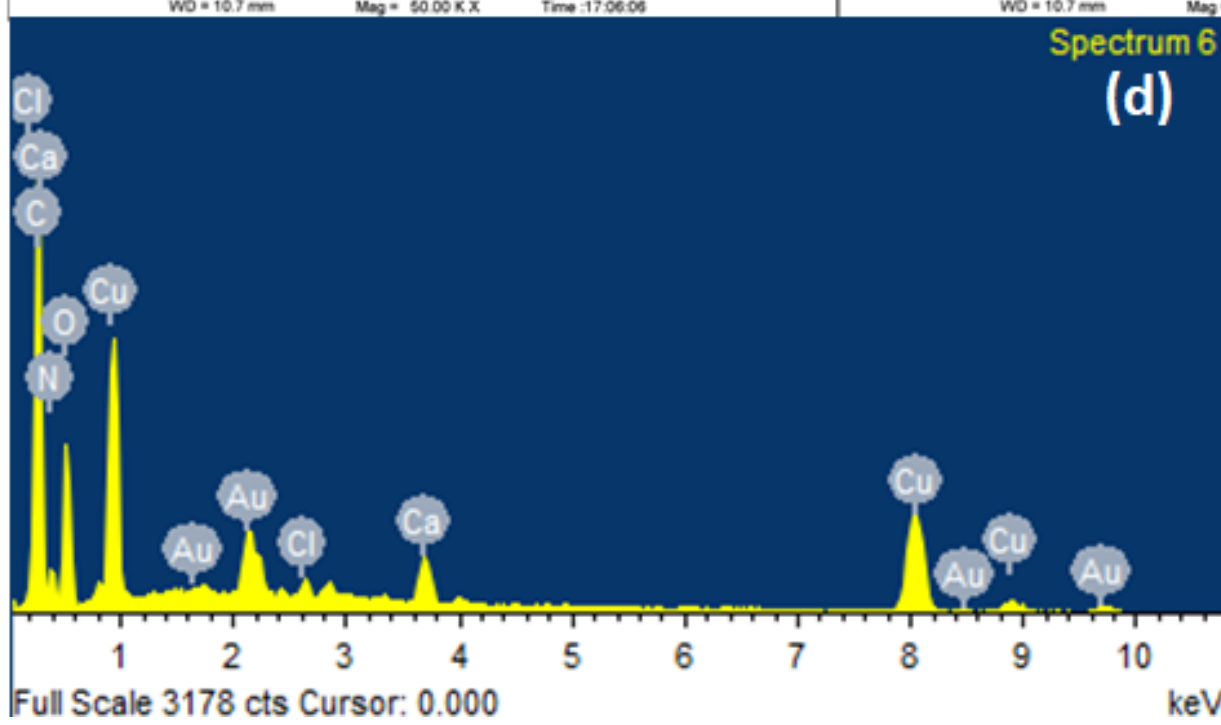

Element	Weight%	Atomic%
C K	44.83	54.14
N K	21.28	22.03
O K	23.48	21.28
Cl K	0.29	0.12
Ca K	0.85	0.31
Cu K	9.28	2.12
Totals	100.00	

Fig. S26. (a, b and c) SEM images of Rbf-Cu-MOF at different magnifications, and (d) EDX spectrum and table show the composition of various elements present in Rbf-Cu-MOF (after durability cycles).

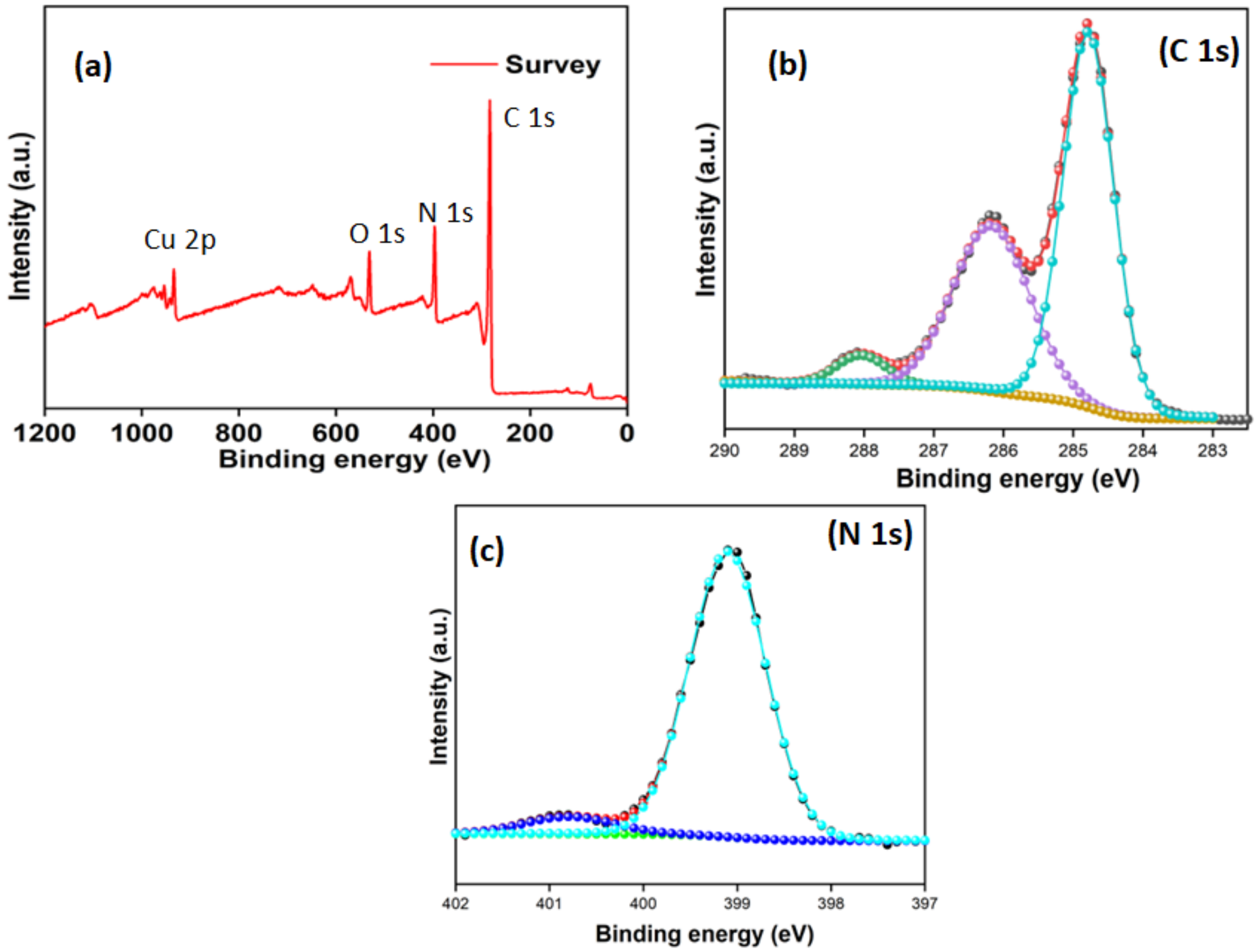

Fig. S27. XPS spectra of Rbf-Cu-MOF (after durability cycles). (a) Survey, (b) C 1s, and (c) N 1s spectra.

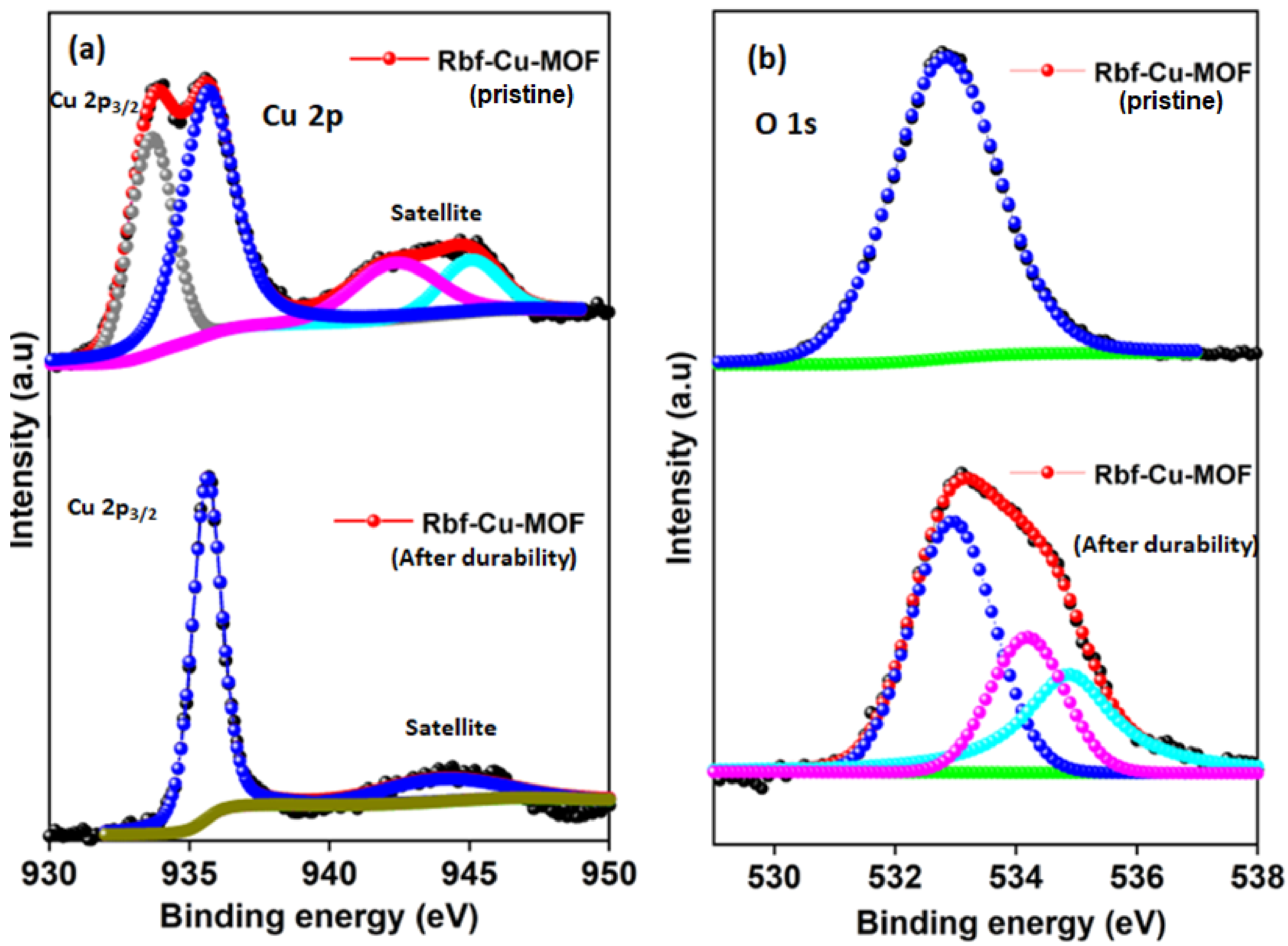

Fig. S28. XPS spectra of Rbf-Cu-MOF (pristine and after durability cycles). (a) Cu 2p and (b) O 1s spectra.

Fig. S29. Proposed mechanism of structural changes in Cu-MOF and Rbf-Cu-MOF under CO_2RR condition.

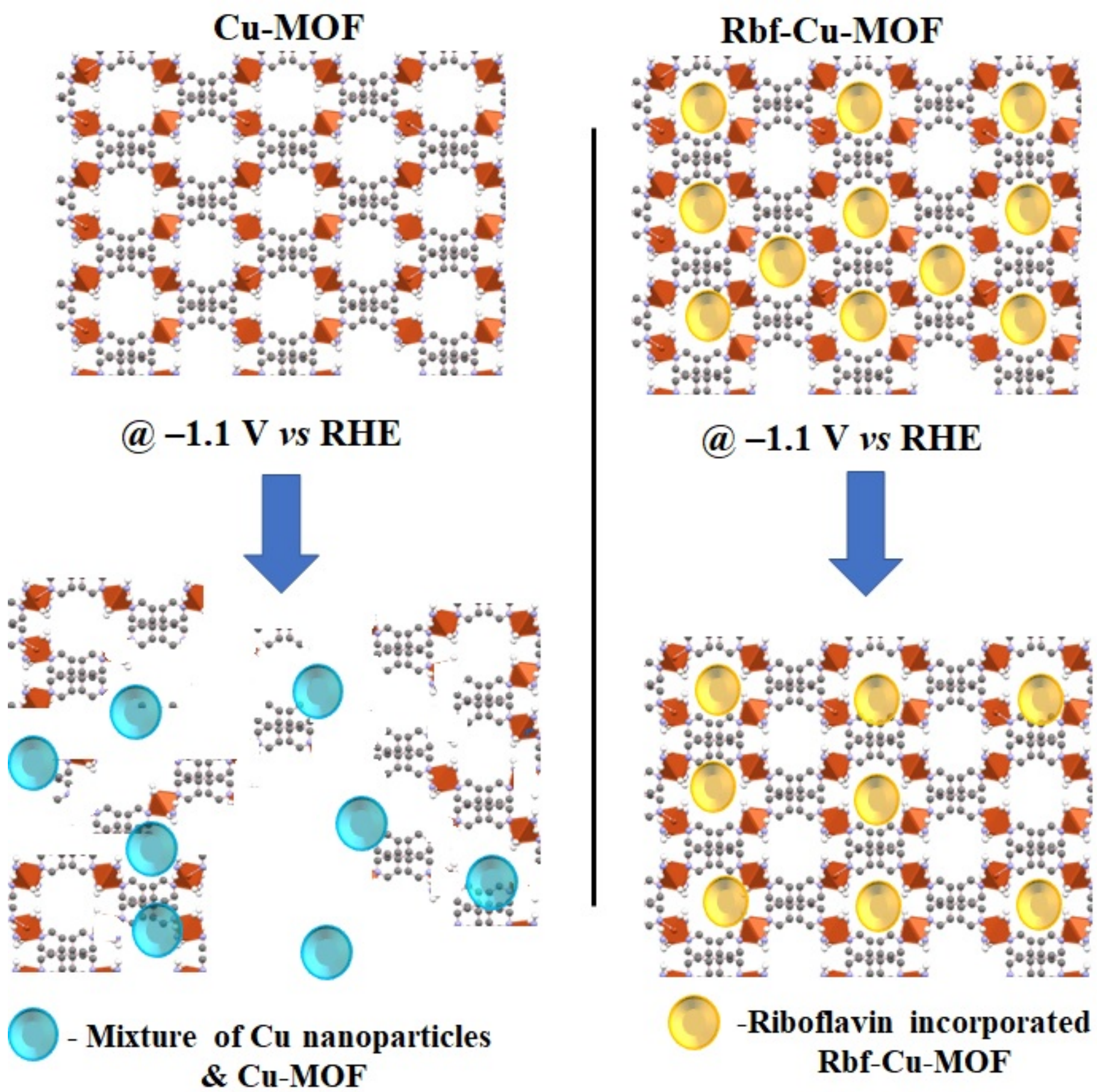

References

1. APEX3 "Program for Data Collection on Area Detectors" BRUKER AXS Inc., 5465 East Cheryl Parkway, Madison, WI 53711-5373 USA.

2. TWINABS, G. M. Sheldrick, "Program for Absorption Correction of Area Detector Frames", BRUKER AXS Inc., 5465 East Cheryl Parkway, Madison, WI 53711-5373 USA.

3. G. M. Sheldrick, SHELXT - Integrated Space-Group and Crystal-Structure Determination, *Acta Crystallogr. A* 2015, **71**, 3-8.

4. O. V. Dolomanov, L. J. Bourhis, R. J. Gildea, J. A. K. Howard and H. Puschmann, OLEX2: A Complete Structure Solution, Refinement and Analysis Program, *J. Appl. Cryst.* 2009, **42**, 339-341.

5. J. Zhang, Q. Chi, S. Dong, and E. Wang, Orientation and Electrocatalysis of Riboflavin Adsorbed on Carbon Substrate Surfaces. *J. Chem. Soc., Faraday Trans.* 1996, **92**, 1913-1920.

6. V. A. Dubskikh, A. A. Kolosov, A. A. Lysova, D. G. Samsonenko, A. N. Lavrov, K. A. Kovalenko, D. N. Dybtsev and V. P. Fedin, A Series of Metal-Organic Frameworks with 2,2'-Bipyridyl Derivatives: Synthesis *vs* Structure Relationships, Adsorption, and Magnetic Studies. *Molecules* 2023, **28**, 2139.
